# Exploring the catalytic activity of –NHSO_3_H functionalized natural asphalt: a sustainable and efficient catalyst for condensation reactions in water†

**DOI:** 10.1039/d5ra00870k

**Published:** 2025-03-26

**Authors:** Sahar Abdolahi, Mohammad Soleiman-Beigi

**Affiliations:** a Department of Chemistry, Faculty of Basic Sciences, Ilam University P.O. Box 69315516 Ilam Iran SoleimanBeigi@yahoo.com m.soleimanbeigi@ilam.ac.ir

## Abstract

Herein, we report the synthesis of natural asphalt sulfamic acid (NA-NHSO_3_H) *via* functionalization of natural asphalt (NA). The –SO_3_H groups on the surface of natural asphalt function as acid catalytic sites. This configuration not only enhances the acidity but also increases the surface area available for catalytic activity, making NA-NHSO_3_H a promising candidate for various acid-catalyzed reactions. The synthesized catalyst was characterized using Fourier Transform Infrared (FT-IR) spectroscopy, Thermogravimetric Analysis (TGA), Scanning Electron Microscopy (SEM), Transmission Electron Microscopy (TEM), Energy Dispersive X-ray (EDX) and X-ray mapping analysis. The catalyst in question has been previously explored for its role in synthesizing various heterocyclic compounds such as polyhydroquinolines, tetrahydrobenzo[*b*]pyran, and 3,4-dihydropyrimidine-2(1*H*)-one/thiones. The NA-NHSO_3_H demonstrated good to excellent yields in the Knoevenagel and Claisen–Schmidt condensations in water at room temperature, indicating its efficiency in these reactions. Conducting these reactions at room temperature and in water can be particularly advantageous, as it enhances safety and sustainability by minimizing the use of hazardous solvents and heat. Conducting these reactions in water at room temperature in the presence of a reusable catalyst aligns with green chemistry principles, which favor the use of benign solvents and milder conditions. This enhances the appeal of NA-NHSO_3_H for industry and academia by reducing environmental impact and energy usage. Furthermore, the catalyst's heterogeneity was evidenced by its excellent reusability and results from hot-filtration tests.

## Abstract 2

Natural asphalt sulfamic acid (NA-NHSO_3_H) was synthesized through the functionalization of natural asphalt (NA). The catalytic performance of NA-NHSO_3_H was evaluated in Knoevenagel and Claisen–Schmidt condensation reactions under mild conditions.

## Graphical abstract

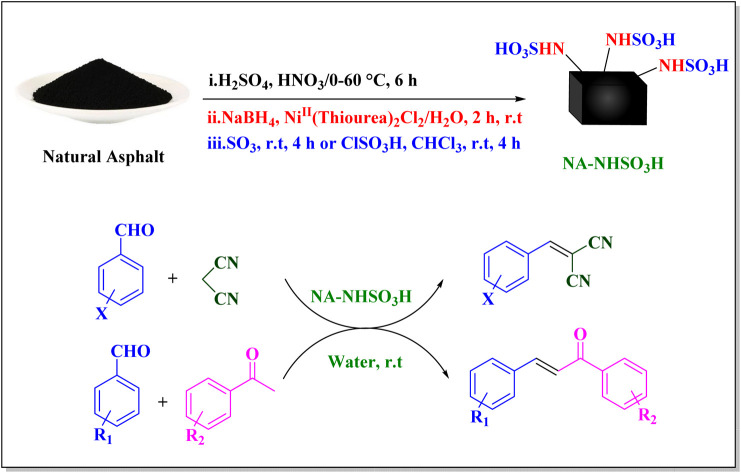

## 1. Introduction

Green chemistry plays a vital role in promoting sustainability within the chemical industry. By focusing on principles like minimizing hazardous substances and reducing waste, it aims to create processes that are not only efficient but also environmentally friendly. The ongoing research into new catalysts and synthesis methods is essential for advancing green chemistry. Heterogeneous Brønsted acids are particularly interesting because they offer several advantages over traditional liquid acids, such as easier separation from products, reusability, and a reduced risk of corrosion. These solid acid catalysts can enhance reaction efficiency and help in achieving higher selectivity, which aligns perfectly with the goals of green chemistry. The development of these solid catalysts and other innovative approaches is crucial for the transition to a more sustainable chemical production process.^1,2^

Carbon nanoallotropes such as fullerene, carbon nanotubes (CNTs), carbon nanofibers (CNFs), graphene and graphene oxide (GO) have attracted significant interest in catalysis due to several beneficial properties including surface chemistry, chemical stability, high surface area, electrical conductivity, and cost-effectiveness. These characteristics make carbon nanomaterials promising candidates for a range of catalytic processes, including fuel cells, environmental remediation, and various organic transformations.^3,4^ The carbon nanoallotropes share a common structural feature – they are all primarily composed of sp^2^-hybridized carbon atoms arranged in a hexagonal network. This shared structural motif is what allows them to be considered part of the same general group or family of carbon nanomaterials. Despite their common hexagonal carbon framework, these nanoallotropes can exhibit quite different properties and behaviors due to factors like their dimensionality, size, and topology. While carbon nanoallotropes like graphene, carbon nanotubes, and fullerenes do share some fundamental properties such as electrical conductivity, mechanical strength, chemical reactivity, and optical characteristics, they differ significantly in terms of their dispersibility in organic solvents. While the raw materials for fullerene synthesis, such as graphite, are inexpensive and abundant, the methods to produce fullerenes often face issues that can make the process costly and environmentally burdensome. The main disadvantages of fullerene preparation methods are low yields, environmental impacts, cost implications, and complicated isolating and purifying. While CNT preparation techniques have advanced significantly, the presence of metallic and amorphous carbon, impurities and synthesis method limitations continues to be a major challenge. The CNFs can indeed be synthesized using methods that are quite similar to those employed for CNTs, particularly through chemical vapor deposition (CVD) and catalytic plasma-enhanced chemical vapor deposition (C-PECVD) techniques. CNFs typically have a more varied structure compared to CNTs. They can be either relatively straight or convoluted and often feature a larger diameter than CNTs. CNFs may also have a more significant presence of graphene-like planes. CNTs, on the other hand, are characterized by their cylindrical nanostructure and can exhibit specific chiralities (which affect their electrical properties). Different preparation methods mentioned have varied efficiencies and effectiveness in purifying CNTs, and each method has its own set of associated costs and scalability challenges. The simultaneous formation of multilayered structures during the production of graphene is indeed a significant hurdle. Isolating pure monolayers requires advanced techniques like chemical vapor deposition (CVD) or liquid-phase exfoliation, which can be intricate and costly. Additionally, the small size of the graphene nanoplatelets, often less than 1 μm, can limit their application in certain fields where larger surfaces are required for optimal performance, such as in composites or conductive materials. This duality of producing high-quality graphene while dealing with size constraints and purity issues poses a complex challenge for researchers and manufacturers. GO is primarily produced by oxidizing graphite to introduce functional groups, which increases its solubility and processability in various solvents. The methods of Brodie, Staudenmaier, and Hummers & Offeman are the most widely cited approaches for synthesizing GO. Both common methods for producing graphene from graphite (direct liquid exfoliation and GO reduction) are challenging due to high solvent consumption, low yield in terms of usable graphene, and time-consuming processes.^3,5–8^

Terrestrial biomass, also known as a carbon source, can be used as a renewable feedstock for the production of biofuels and valuable chemicals. Lignocellulosic biomass is indeed the most abundant organic carbon source on Earth, comprising a significant portion of the world's biomass. Lignocellulosic biomass is widely available from agricultural residues, wood, and dedicated energy crops, making it a promising feedstock. Using lignocellulosic biomass can contribute to a circular economy by utilizing waste materials and reducing reliance on fossil fuels. This source can be converted into a variety of products, including bioethanol, biobutanol, phenolic compounds, and other specialty chemicals.^9,10^

Ongoing research continues to optimize carbon-based materials properties for specific catalytic applications. There are several Brønsted acid catalysts, including *p*-toluene sulfonic acid (*p*-TsOH),^11^ silica-sulfuric acid (SSA),^12^ PPF-SO_3_H,^13^ sulfonic MCM-41 (MCM-SO_3_H),^14^ chitosan (CS)-derived magnetic solid acid catalyst (CS-Fe_3_O_4_@SO_3_H),^15^ organosilane sulfonated graphene oxide (SSi-GO)^16^ and cellulose sulfuric acid (CSA),^17^ which have advantages such as reusability and recyclability, good yield, short reaction time, and easy separation. However, they suffer from expensive starting materials and reagents, complicated synthesis, low catalytic activity and product separation with toxic solvents.

Discovering new supports for Brønsted acid catalysts is essential for overcoming current limitations and advancing the field of catalysis. The discovery and utilization of natural resources have profoundly transformed global energy. Natural asphalt (NA) is a naturally occurring substance primarily composed of hydrocarbons. It can be found in the form of deposits, often in locations like natural seeps or tar pits. When mined and processed, it typically has a shiny, black appearance but can break down into a powdery consistency under certain conditions. NA is known for its durability and resistance to water, making it a valuable resource in many industries. Unlike carbon nanomaterials, this natural material is available, non-toxic and cheap. It has high carbon content and can be an excellent candidate as a catalyst support in organic reactions.^18–20^ NA is characterized by its high melting point, typically between 180 to 245 °C, which makes it suitable for various applications requiring thermal stability. Research indicates that NA exhibits high solubility in several organic solvents, including toluene, xylene, and carbon disulfide. NA consists of hydrocarbons, which can include polycyclic aromatic structures, saturated hydrocarbons and functional groups. The structure of NA is complex, but its analysis shows that it contains 70–80% of carbon, 15% of hydrogen and other elements including traces of nitrogen, sulfur, oxygen and various metals.^19–21^

Furthermore, it is eco-friendly and can enhance the sustainability of chemical processes. The functional groups present on the surface of NA can significantly influence its interaction properties, making it a valuable substrate for supported catalyst. It enhancing catalytic efficiency and selectivity in various chemical reactions. This attribute is particularly advantageous in processes like heterogeneous catalysis, where effective dispersion and stabilization of active sites are crucial.^22^

Traditional liquid-acid catalysts, like sulfuric acid (H_2_SO_4_), hydrochloric acid (HCl), hydrobromic acid (HBr) and trifluoroacetic acid (CF_3_COOH) often provide high efficiency in various chemical reactions, particularly in processes. Indeed, the challenges associated with liquid-acid catalysts in homogeneous reactions can lead to significant environmental and economic concerns. The difficulties in separation and recovery often result in large quantities of non-recyclable acid waste, which not only increases disposal costs but also poses environmental risks. To mitigate these issues, several strategies can be applied, including use of solid acid catalysts, alternative reaction conditions, immobilization techniques, continuous flow systems, and continuous flow systems. Solid acid catalysts gained significant attention in recent years due to their numerous advantages such as non-toxicity, reusability, lower costs, milder reaction conditions, environmental benefits, simple handling, stability, and diverse applications in various chemical reactions. They facilitate reactions by providing a pathway with lower activation energy, thus increasing reaction rates. The ability to recover and reuse solid catalysts significantly contributes to the principles of green chemistry by minimizing waste and reducing the need for additional resources. Solid catalysts often allow for easier separation from reaction mixtures compared to homogeneous catalysts, leading to less environmental impact. Their reusability not only enhances cost-effectiveness but also decreases the overall energy and material consumption associated with chemical processes. This aligns well with the goals of sustainable development in chemical manufacturing.^2,23–26^

The promotion of simple and neat protocols in synthetic chemistry aligns closely with the principles of green chemistry, which emphasize sustainability and minimizing environmental impact. The evolution of synthetic chemistry is increasingly focusing on atom-economy, cost-effectiveness, and environmental sustainability. This shift is driven by the need to minimize waste and reduce the environmental impact of chemical processes, which is crucial for both industrial applications and academic research. Accordingly, Claisen–Schmidt and Knoevenagel condensations have garnered significant interest due to their ability to produce target products in a single synthetic step. This approach eliminates the need to separate intermediates, which contributes to reduced energy consumption, shorter reaction times, and minimized waste materials. As a result, these methods promote environmentally friendly processes in organic synthesis.^27–30^

The Knoevenagel condensation is a key reaction in organic synthesis, valued for its efficiency in forming carbon–carbon double bonds (C=C). The products of the Knoevenagel condensation serve as valuable organic intermediates that can be further transformed into a wide variety of natural compounds with significant medicinal and physical properties. In industry, Knoevenagel products are essential intermediates in the synthesis of pharmaceuticals, agrochemicals, and fine chemicals. The ongoing research into the Knoevenagel condensation reflects both its fundamental importance in organic synthesis and its adaptability to modern sustainable chemistry practices. The continuous exploration in this area is likely to lead to more efficient, cost-effective, and environmentally friendly synthetic methodologies.^31^

The Claisen–Schmidt (CS) condensation is a specific type of cross-aldol condensation, especially for forming α,β-unsaturated carbonyl compounds. This reaction is pivotal in organic synthesis for creating α,β-unsaturated carbonyl compounds, which are important in various chemical applications. Many drugs and drug precursors are synthesized using CS condensation. Compounds such as flavonoids and their derivatives, which often exhibit anti-inflammatory, antioxidant, anti-viral, and anti-cancer properties, are commonly produced *via* this reaction. Chalcones, a class of compounds derived from CS condensation, are extensively studied for their potential therapeutic benefits. The reaction is utilized in the synthesis of flavor compounds and food additives, enhancing the sensory attributes of food products. Colorants derived from chalcones and flavonoids are also important in food and beverage applications for enhancing color without synthetic dyes. The CS reaction is valuable in the production of specialty chemicals used in perfumes and fragrances.^32,33^

The CS reaction can be catalyzed by bases or acids, and the choice of catalyst can significantly influence the reaction conditions and outcomes. However, this process is plagued by reverse reactions and side reactions that lower overall yields. In the past few decades, various metal(ii) ion complexes such as Yb(OTf)_3_, RuCl_3_, FeCl_3_, Cp_2_ZrH_2_, TMSCl/NaI, KF-Al_2_O_3_, SmI_3_, InCl_3_, BF_3_·OEt_2_, TiCl_3_(SO_3_CF_3_) and BMPTO have been introduced as catalysts. However, the yields were not satisfactory (<38%). However, in most cases, good yields are achieved at high temperatures, but these conditions can be accompanied by challenges such as longer reaction times and complex and tedious purification procedures.^34^

Herein, the NA-NHSO_3_H catalyst is being applied for Knoevenagel and Claisen–Schmidt condensations. These condensation reactions are carried out in water as the solvent and at room temperature. The use of NA-NHSO_3_H as a solid acid catalyst in the Knoevenagel and Claisen–Schmidt condensations is an interesting approach, as it combines the advantages of NA (easy separation and recovery) with the catalytic activity of the –SO_3_H groups. This type of heterogeneous, recoverable catalyst can potentially offer improved efficiency, sustainability, and recycling capabilities compared to traditional homogeneous catalysts.

## 2. Experimental

### 2.1. Materials and instruments

All solvents and chemicals utilized in this study were obtained from Sigma-Aldrich and Merck chemical companies, and NA was obtained from the natural bitumen mines in west of Iran. Fourier Transform Infrared (FT-IR) spectroscopy was conducted using a VERTEX 70 FT-IR spectrometer from Bruker, Germany. The samples were prepared as KBr pellets for analysis. Scanning Electron Microscopy (SEM) imaging was performed using a TESCAN MIRA III Field Emission Scanning Electron Microscope (FE-SEM) from the Czech. Energy-dispersive X-ray spectroscopy (EDX) mapping was performed using a TESCAN instrument from the Czech to analyze the elemental composition of the samples. Additionally, the nitrogen adsorption–desorption isotherms were measured at 77 K using the BET method with a Micromeritics Asap 2020 instrument from the USA. Thermogravimetric analysis (TGA) was conducted using a NETZSCH TGA instrument, measuring temperature ranges from 25 °C to 800 °C to assess the thermal stability of the samples. Transmission Electron Microscopy (TEM) imaging of NA-NHSO_3_H was carried out using a Philips EM 208S TEM to investigate the morphology and size of the particles. ^1^HNMR and ^13^CNMR spectra was performed on Bruker Avance DPX-400 and DPX-500 spectrometers. Chemical shifts were reported in parts per million (ppm) relative to tetramethylsilane (TMS), which served as the internal standard.

### 2.2. Preparation and characterization of NA-NHSO_3_H

#### 2.2.1. NA-NO_2_ synthesis *via* nitration of NA

A mixture of 17.5 mL of nitric acid and 20 mL of sulfuric acid was prepared in a 250 mL flask and allowed to stand at 0 °C for 15 minutes. Subsequently, 2 g of NA was slowly added to the acid mixture. After 30 minutes, the reaction temperature was raised to 60 °C, and the reaction proceeded for 5 hours. At the conclusion of the reaction, 300 mL of distilled water was added to the mixture. The resulting solution was then filtered, and the residue was washed with water (3 times, 15 mL each wash). Finally, the product was dried in an oven at 80 °C for 3 hours.

#### 2.2.2. NA-NH_2_ synthesis *via* reduction of NA-NO_2_

##### 2.2.2.1. Catalyst preparation for reduction reaction

In a 50 mL flask, 0.02 mol of thiourea was dissolved in a mixture of ethanol and water (20 mL each). The solution was heated to 50 °C to ensure complete dissolution of thiourea. Once dissolved, 0.005 mol of nickel(ii) chloride hexahydrate (NiCl_2_·6H_2_O) was added. The reaction mixture was stirred at 80 °C for 4 hours. After the reaction was complete, the mixture was allowed to stand at room temperature for one week to facilitate the evaporation of ethanol, resulting in the formation of the Ni^II^(Thiourea)_2_Cl_2_ complex.^21^ The product was then washed several times with ethanol to purify it. The final yield of the pure complex was 95%.

##### 2.2.2.2. Convert the –NO_2_ group to –NH_2_

1.0 g of NA-NO_2_ and 1.0 g of Ni^II^(Thiourea)_2_Cl_2_ complex were added to a 100 mL two-neck round-bottom flask. The reaction mixture was stirred for 20 minutes. To manage the production of hydrogen gas (H_2_), the temperature of the reaction was lowered to 0 °C. Then, 0.7 g of sodium borohydride (NaBH_4_) was added to the flask. After 2 hours, the reaction mixture was allowed to stand at room temperature for 24 hours to dry. Ultimately, 0.8 g of product was obtained.

#### 2.2.3. Synthesis of NA-NHSO_3_H

**Scheme 1:**
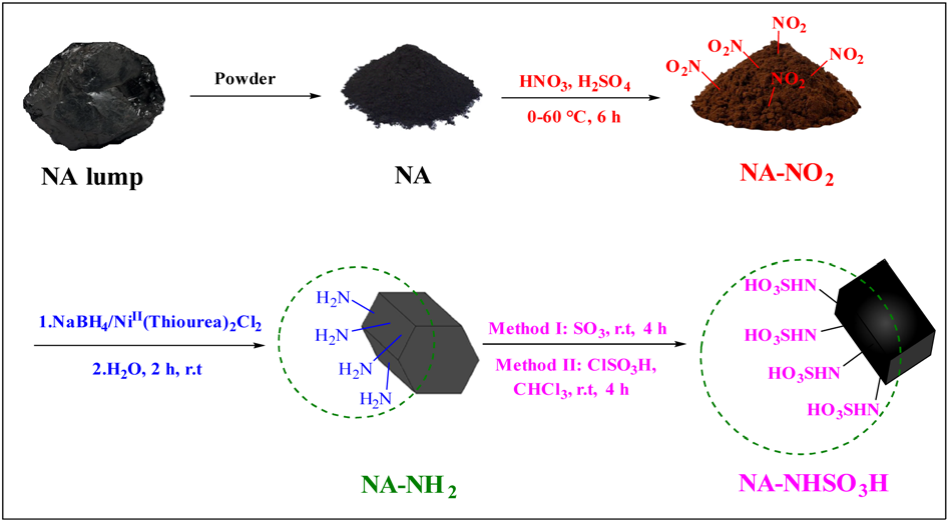
General route for the synthesis of NA-NHSO_3_H.

Two methods were tested to prepare NA-NHSO_3_H (Scheme 1):

Method I: 0.5 g of NA-NH_2_ was added to 5 mL of liquid SO_3_ into a round-bottom flask at 0 °C. The reaction mixture was stirred at room temperature for 4 hours. The reaction mixture, then, was poured to cold water slowly, precipitate was filtered, washed with water and dried. Finally 0.92 g of the product was obtained.

Method II: After pouring 1.5 g of NA-NH_2_ and 15 mL of chloroform into a round-bottom flask, 0.5 mL of chlorosulfonic acid was added dropwise to the mixture for 15 minutes at −5 °C. The reaction was then continued for 4 hours at room temperature.

The reaction mixture, then, was poured to cold water slowly, the resulting precipitate was washed with water and dried. After drying, 0.9 g of the product was prepared.^18^

## 3. Results and discussion

NA-NHSO_3_H was characterized using Fourier transform infrared (FT-IR) spectroscopy, thermogravimetric analysis (TGA), scanning electron microscopy (SEM), transmission electron microscopy (TEM) and energy-dispersive X-ray spectroscopy (EDX) mapping.

### 3.1. Characterization of NA-NHSO_3_H

#### 3.1.1. FT-IR studies

**Fig. 1:**
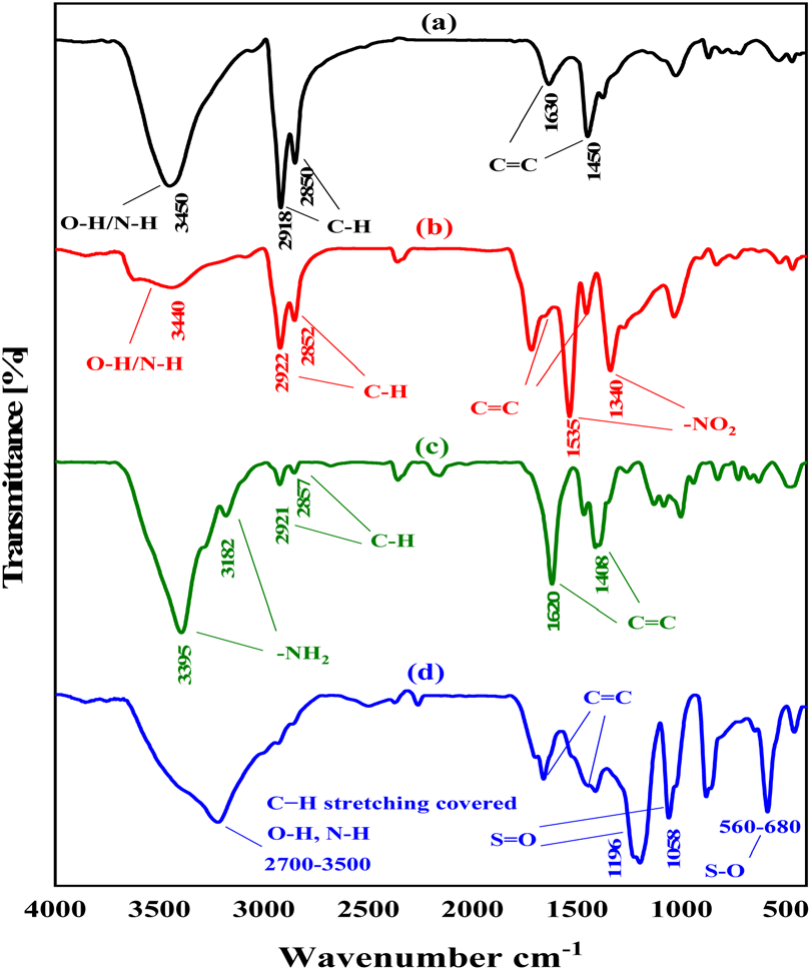
FT-IR spectra of NA (a), NA-NO_2_ (b), NA-NH_2_ (c) NA-NHSO_3_H (d).

Fig. 1 exhibits the FTIR spectrum of NA-NHSO_3_H, which differs from the FT-IR spectra of non-functionalized NA, NA-NO_2_, and NA-NH_2_. The stretching vibrations of the functional groups of the compounds mentioned are summarized in Table 1.

**Table 1:** Stretching vibrations related to functional groups present in NA, NA-NO_2_, NA-NH_2_ and NA-NHSO_3_H

Functional group	NA	NA-NO_2_	NA-NH_2_	NA-NHSO_3_H
C=C aromatic	1450–1630 cm^−1^	1446–1638 cm^−1^	1408–1620 cm^−1^	1426–1635 cm^−1^
C–H aliphatic	2850–2918 cm^−1^	2852–2922 cm^−1^	1857–2921 cm^−1^	Covered
OH/NH	3450 cm^−1^	3440 cm^−1^	Covered	2700–3500 cm^−1^
NO_2_	—	1340–1535 cm^−1^	—	—
NH_2_	—	—	3182–3395 cm^−1^	—
S–O	—	—	—	560–680 cm^−1^
SO_2_	—	—	—	1058–1196 cm^−1^

#### 3.1.2. TGA analysis

**Fig. 2:**
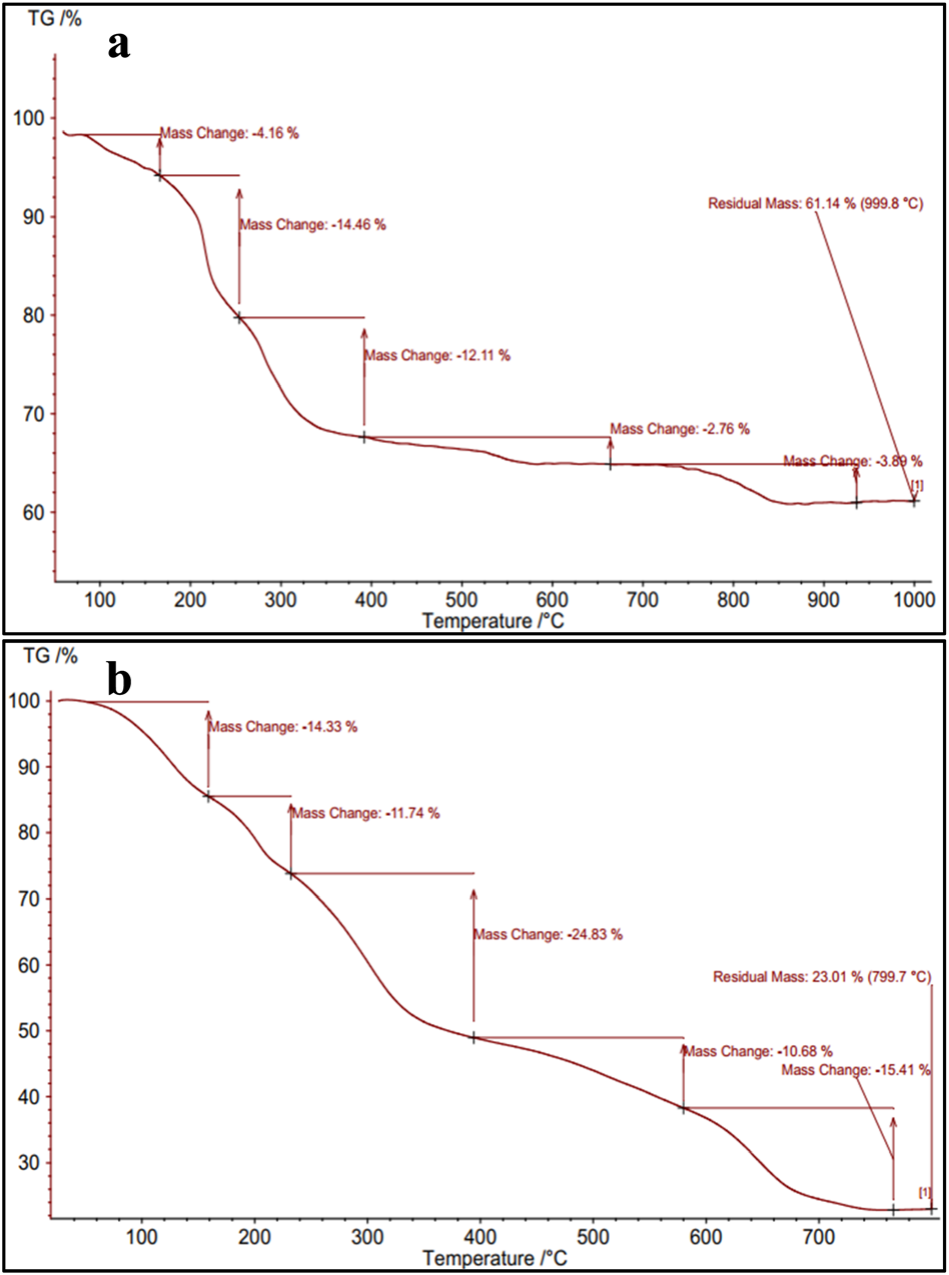
TG diagrams of (a) NA and (b) NA-NHSO_3_H.

Thermogravimetric analysis (TGA) is a valuable technique for assessing the thermal stability of various samples. For this purpose, the thermal stability of NA and NA-NHSO_3_H was evaluated. TG curve in NA-NHSO_3_H typically shows various weight loss steps corresponding to different degradation processes. The initial weight loss (∼22%) below 200 °C likely indicates the removal of water and any residual organic solvents that may be present on the catalyst's surface. This process can affect the overall performance of the catalyst. The ∼25% degradation observed in the 200–400 °C range indicates that organic compounds are undergoing thermal decomposition. The final weight loss of about 23% observed at temperatures between 400–700 °C likely indicates the decomposition of –SO_3_H groups. At elevated temperatures, the –SO_3_H groups may decompose, leading to the release of gases such as sulfur dioxide and water, resulting in the observed weight loss. This thermal behavior is typical in materials containing –SO_3_H due to the stability of these groups at lower temperatures and their susceptibility to degradation at higher temperatures. But in NA, only 34% of the material has decomposed when subjected to temperatures ranging from 25 to 700 °C. This is while 70% of NA-NHSO_3_H was decomposed. These findings confirm that the sulfur dioxide functional groups were removed and the catalyst was successfully synthesized (Fig. 2).

#### 3.1.3. Scanning electron microscopy (SEM) studies

**Fig. 3:**
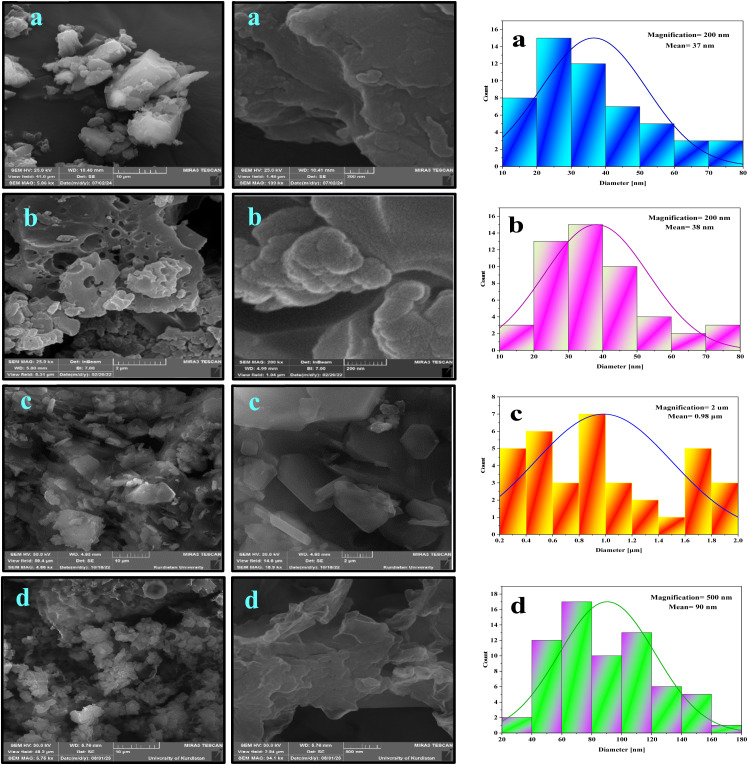
SEM images of NA (a), NA-NO_2_ (b), NA-NH_2_ (c) and NA-NHSO_3_H (d) with their histograms.

Scanning electron microscopy (SEM) was used for NA, NA-NO_2_, NA-NH_2_ and NA-NHSO_3_H, as shown in Fig. 3a–d. SEM is an excellent technique for analyzing microstructural features, as it provides high-resolution images that can reveal the morphology, texture, and shape of materials. SEM images of NA (Fig. 3a) and NA-NO_2_ (Fig. 3b) indicate that the nanoparticles are predominantly spherical and their average diameter is 37 and 38 nm, respectively. On the contrary, NA-NH_2_ (Fig. 3c) does not have a spherical shape; rather, its shape consists of hexagonal crystals. The average particle size of 0.98 μm indicates that its hexagonal particles are quite uniform. The particle size reduction from 0.98 μm to 90 nm upon stabilization –NHSO_3_H groups suggests that the functional groups have a significant effect on the morphology of the particles, likely enhancing their dispersion or preventing agglomeration. The fact that NA-NHSO_3_H forms from cubic crystals suggests that the –NHSO_3_H groups play a role not just in size reduction, but also in maintaining a uniform particle distribution (Fig. 3d). This uniformity can be crucial for various applications like catalysis or adsorption.

#### 3.1.4. Transmission electron microscopy (TEM) studies

**Fig. 4:**
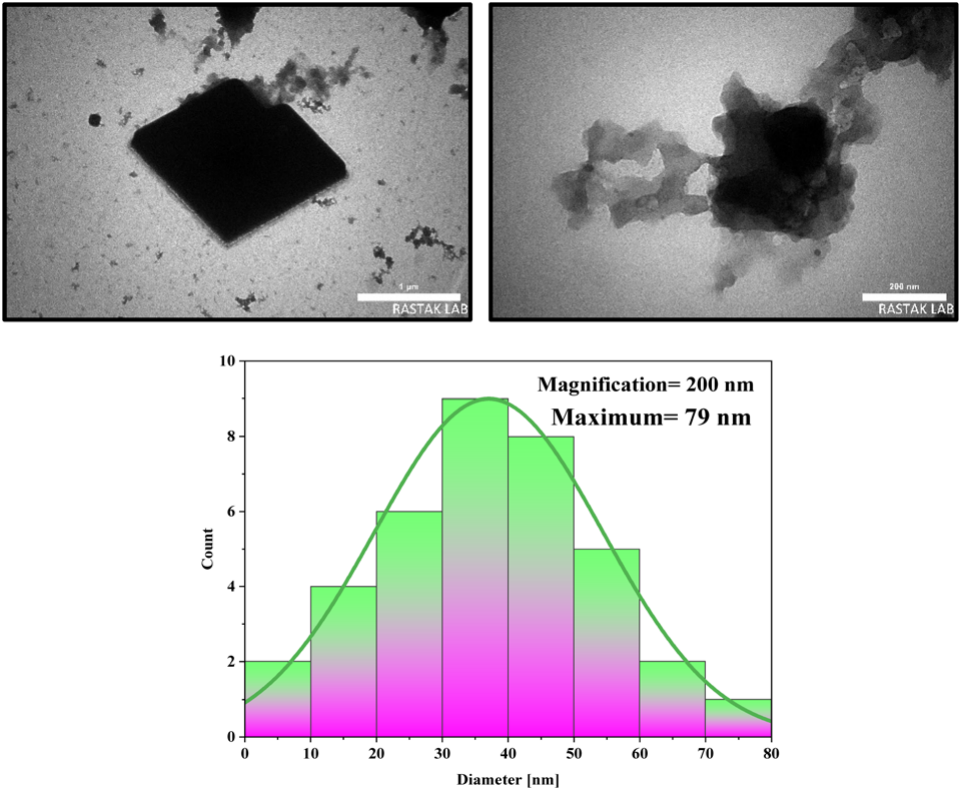
TEM images of NA-NHSO_3_H at different magnifications and its histogram.

Transmission Electron Microscopy (TEM) is an effective technique for analyzing the morphology and particle size of materials. The TEM images effectively confirm that the catalyst consists of cubic crystals, which can influence its properties and reactivity. The observation that the crystals are interconnected in a chain network suggests enhanced catalytic activity, likely due to increased surface area and improved electron transfer between particles. In addition, TEM images demonstrated that the prepared NA-NHSO_3_H has a regular and ordered structure. An ordered structure in a catalyst often indicates better performance in catalytic reactions, as it can enhance factors like surface area, stability, and interaction with reactants. Furthermore, TEM images demonstrate hydrogen bonding between cubic crystals of NA-NHSO_3_H, and these observations are correlated with the SEM images of Fig. 3d, indicating that hydrogen bonds significantly contribute to the stability or formation of the cubic crystal structure in NA-NHSO_3_H. The histogram data showing a maximum particle size of 79 nm is significant, especially as particle size often plays a crucial role in catalytic activity. The TEM results are consistent with the SEM analysis, indicating that the size and shape of the crystals are consistent between the two techniques (Fig. 4).

#### 3.1.5. EDX and elemental mapping analysis

**Fig. 5:**
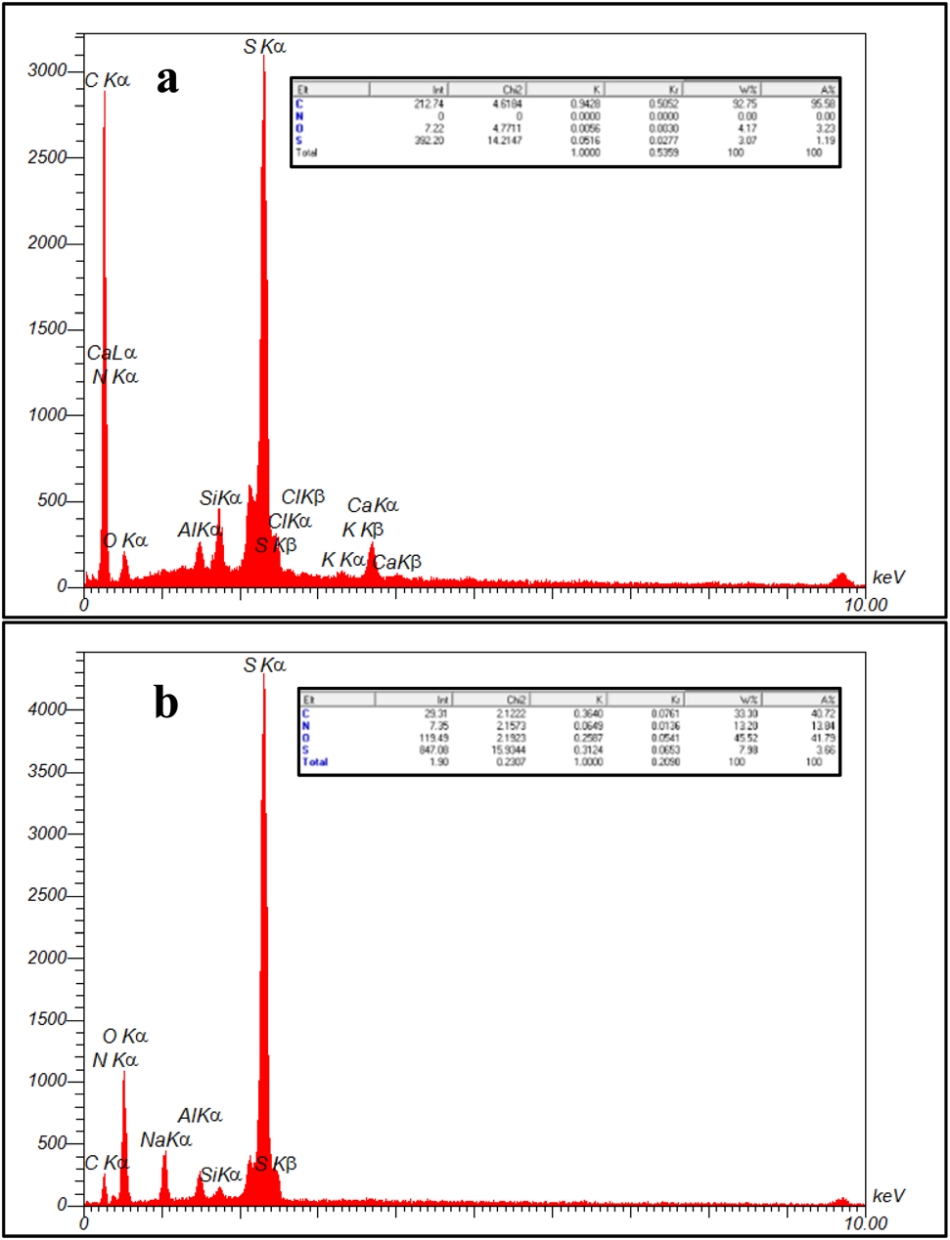
EDX spectrum of (a) NA and (b) NA-NHSO_3_H.

**Fig. 6:**
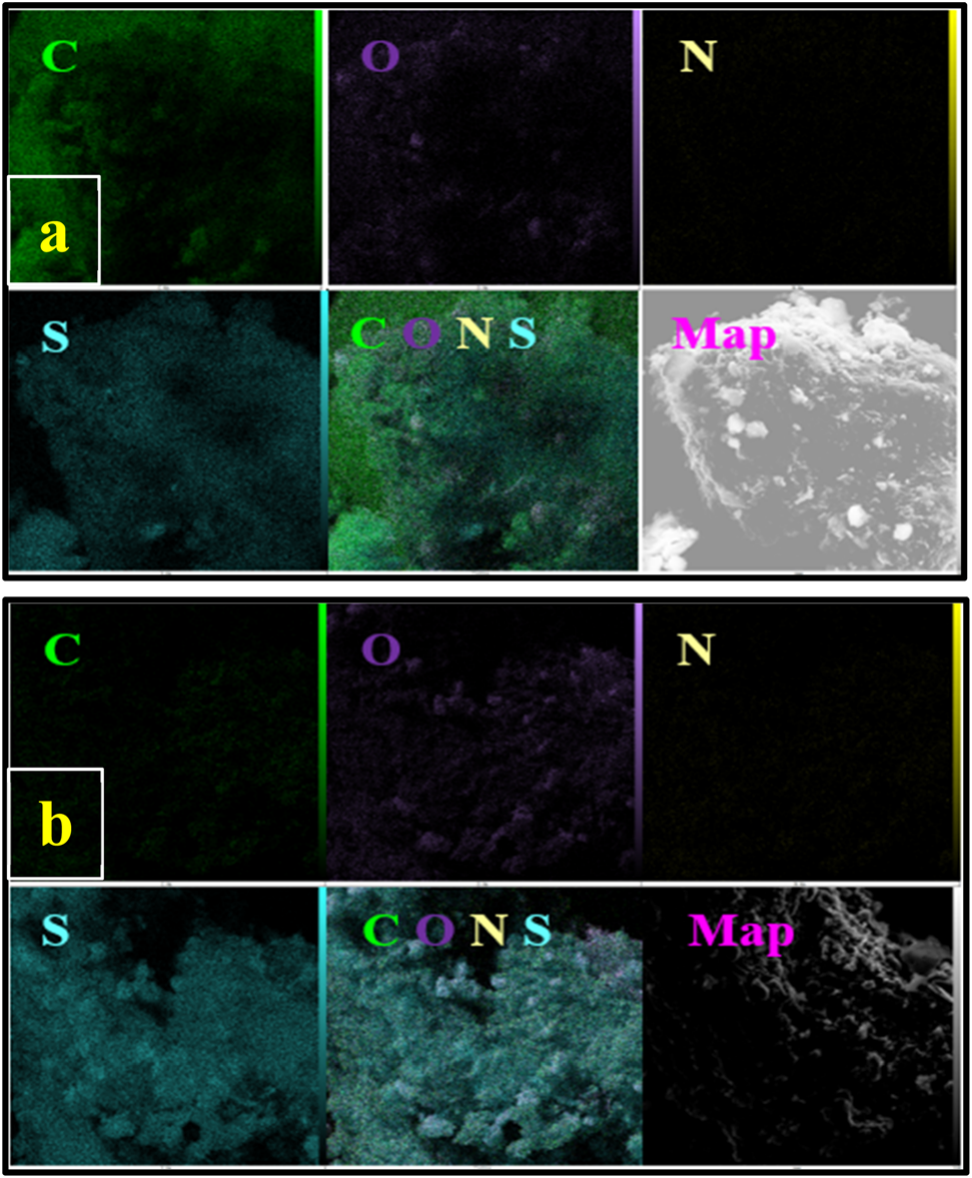
Elemental mapping of (a) NA and (b) NA-NHSO_3_H.

EDX analysis was performed to investigate the elemental characteristics and mapping of NA, NA-NHSO_3_H in Fig. 5a, b and 6a, b, respectively. The EDX result showed a good elemental distribution was obtained in NA and NA-NHSO_3_H (Fig. 5a and b). Good elemental distribution indicates that the different components are well-mixed, which is crucial for ensuring uniform properties in materials. Additionally, the map images of the elements (C, S, N and O) in NA and NA-NHSO_3_H have suitable dispersion and homogeneity (Fig. 6a and b). The mention of suitable dispersion and homogeneity in the elemental map images for C, S, N, and O in NA and NA-NHSO_3_H suggests that these elements are evenly distributed throughout the samples. The comparison of EDX analysis and elemental mapping in NA and NA-NHSO_3_H confirms that the content of sulfur in NA-NHSO_3_H is increased, which can be due to the successful immobilization and incorporation of the –SO_3_H group onto the NA support. Also, this increase in the amount of sulfur is clearly visible in the distribution map of the elements. The increase in sulfur suggests that the functionalization process was effective, enhancing the chemical properties and possibly the catalytic activity of NA-NHSO_3_H onto the NA support. Combining EDX and elemental mapping provides a comprehensive understanding of the elemental composition and distribution of NA and NA-NHSO_3_H, which is invaluable for characterizing materials in various scientific fields (Fig. 5 and 6).

The presence of metal sulfides and SiO_2_ in NA and their subsequent removal in an acidic environment can provide valuable insights into the catalyst's performance and the stability of the materials involved. The acidic environment likely promotes the dissolution or transformation of the metal sulfides and SiO_2_. Understanding the specific reactions that occur in NA-NHSO_3_H could help in optimizing the catalyst further. The removal of these materials might enhance the catalytic properties of the remaining structure, possibly by increasing the availability of active sites or improving the overall morphology of the catalyst.

### 3.2. Catalytic studies

#### 3.2.1. Knoevenagel condensation

The Knoevenagel condensation reaction involves the condensation of aldehydes with active methylene compounds. This reaction leads to the formation of α,β-unsaturated carbonyl compounds, which are valuable intermediates in various synthetic pathways. The products of Knoevenagel condensation, specifically the α,β-unsaturated products or synthesized alkenes, have indeed been widely utilized as intermediates in various fields such as of fine chemicals and pharmaceuticals, functional polymers, cosmetics and perfumes, natural products and synthesis of heterocyclic compounds of biological. The investigation of the catalytic performance of the NA-NHSO_3_H system in Knoevenagel condensation is quite interesting, especially given its implications for developing new synthetic methods. This reaction is pivotal in organic synthesis, particularly for creating compounds that have potential biological and medicinal applications.

For this purpose, the condensation of malononitrile with 4-chlorobenzaldehyde was used as a model reaction. Then, different reaction conditions were screened to optimize the process. The results of the optimization experiments are summarized in Table 2. The results showed that 10 mg of catalyst, water and room temperature were selected as the best reaction conditions among different amounts, solvents and temperatures (Table 2, entry 4)

**Table 2:** Optimization of Knoevenagel condensation *via* the reaction between 4-chlorobenzaldehyde and malononitrile^a^

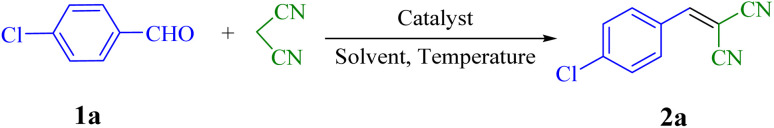					
Entry	NA-NHSO_3_H (mg)	Solvent	Temperature (°C)	Time (min)	Yield^b^ (%)
1	—	Water	r.t.	270	36
2	20	Water	r.t.	20	90
3	15	Water	r.t.	18	93
**4**	**10**	**Water**	**r.t.**	**18**	**99**
5	5	Water	r.t.	30	91
6	10	Ethanol	r.t.	84	86
7	10	Water : ethanol (1 : 1)	r.t.	55	88
8	10	Water : ethanol (2 : 1)	r.t.	35	90
9	10	Acetonitrile	r.t.	300	48
10	10	Water	50	28	92
11	10	Water	80	34	90
12	10	Water	r.t.	150	31^c^

a Reaction conditions: 4-chlorobenzaldehyde (1 mmol, 140 mg), malononitrile (1.05 mmol, 69 mg), NA-NHSO_3_H (mg) and solvent (1 mL).

b Isolated yield.

c The reaction catalyzed by NA-NH_2_.

The observed decrease in reaction yield with increasing temperature can be attributed to the potential hydrolysis of nitrile groups within the product. This hydrolysis, occurring at higher temperatures, generates by-products and intermediates that interfere with the intended Knoevenagel reaction pathway. Furthermore, competing side reactions, including Diels–Alder and hetero-Diels–Alder additions, contribute to the formation of various by-products.^35^ These side reactions effectively compete with the formation of the desired Knoevenagel product, leading to a dilution of its concentration and a subsequent reduction in both the overall yield and reaction rate (Tables 2, 4 and 5). In many chemical reactions, especially reactions involving organic substrates, the strength of the acid can affect the reaction rate. Acids can act as catalysts and lower the activation energy by protonating the reactants. This causes the reaction to proceed more quickly. The pH scale is logarithmic and measures the concentration of hydrogen ions (H^+^) in a solution. As the temperature rises, the ionization of water increases, leading to a higher concentration of H^+^ ions. This can cause the pH value to decrease (become more acidic), even in pure water (eqn (1)).

1 pH = −log H^+^

To investigate the effect of temperature on the reaction rate and acidity, a glass electrode was used. Three different temperatures (r.t., 50 °C, 80 °C) were investigated for the reaction of model 2a. First, 10 mg of the catalyst was dispersed in 5 mL of deionized water at room temperature. Then, the pH at room temperature, 50 and 80 °C showed the values of 2.10, 2.21 and 2.33, respectively. The effect of temperature on the reaction rate was also evaluated for reactions 4a and 6a at r.t., 45, and 70 °C, respectively. The results indicated that for reaction 4a, pH values of 2.11, 2.23, and 2.36 were obtained, respectively. Furthermore, for reaction 6a, values of 2.11, 2.24, and 2.37 were measured, respectively. The observed relationship between increasing temperature, decreasing pH, increasing acid strength, and accelerating reaction rate is a coherent and well-supported pattern in chemistry. As temperature rises, changes in high pH cause a decrease in acid strength, which ultimately slows down the reaction (Tables 2, 4 and 5).

The relationship between the Nernst equation, acid strength, reaction rate, and the effect of decreasing temperature is examined. The Nernst equation describes how the potential of an electrode (*E*) changes with the concentration of the reactants and products involved in an electrochemical reaction (eqn (2)).^36^2
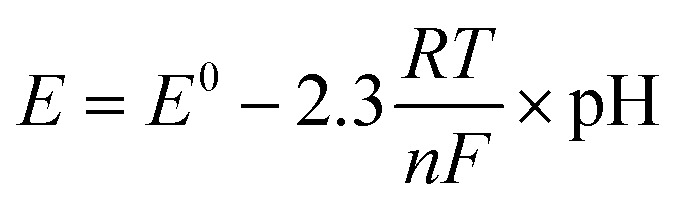
where *E*: cell potential under non-standard conditions. *E*^0^: standard cell potential. *R*: ideal gas constant (8.314 J mol^−1^ K^−1^). *T*: temperature (in Kelvin). *n*: number of moles of electrons transferred in the cell reaction. *F*: Faraday's constant (96 485C mol^−1^).

The acid strength of a solution is determined by its pH. Acidic solutions have a lower pH and a higher concentration of hydrogen ions (H^+^). A decrease in temperature directly reduces the magnitude of the (*RT*/*nF*) term. Smaller (*RT*/*nF*) means it will have less effect on the cell potential (*E*). In other words, the cell potential becomes less sensitive to changes in concentration as the temperature decreases. In addition, if the acid strength decreases (pH increases, [H^+^] decreases), it will also decrease the cell potential (*E*). A lower cell potential generally translates to a slower reaction rate. The driving force for the electron transfer is reduced (Tables 2, 4 and 5).

**Scheme 2:**
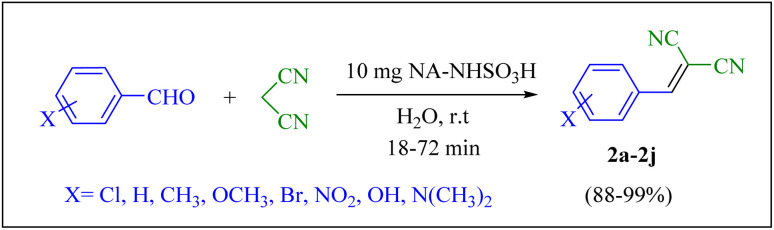
Knoevenagel condensation of malononitrile with aromatic aldehydes catalyzed by NA-NHSO_3_H.

The generality of the catalytic system was evaluated by reacting different aldehydes (both electron-donating and electron-withdrawing substituted aromatic aldehydes) with malononitrile (Scheme 2). According to the results, the desired products were obtained in satisfactory to excellent yields, suggesting a high degree of efficiency in the reaction process (Table 3).

**Table 3:** NA-NHSO_3_H catalyzed the Knoevenagel condensation in water at room temperature^a^

Entry	Aldehyde	Product	Time (min)	Yield (%)	Mp (°C) measured	Mp (°C) literature
1	4-ClC_6_H_4_CHO	2a	18	99	160–162	162–164 (ref. 37)
2	C_6_H_5_CHO	2b	24	97	80	80–82 (ref. 37)
3	4-MC_6_H_4_CHO	2c	38	94	128–130	130–133 (ref. 37)
4	4-MeOC_6_H_4_CHO	2d	54	91	116–118	114–116 (ref. 37)
5	3,4-(MeO)_2_C_6_H_3_CHO	2e	64	88	130–132	132–135 (ref. 38)
6	4-BrC_6_H_4_CHO	2f	72	89	150–152	153–156 (ref. 38)
7	3-O_2_NC_6_H_4_CHO	2g	45	93	102–104	106–108 (ref. 37)
8	4-O_2_NC_6_H_4_CHO	2h	40	95	158–160	159–161 (ref. 30)
9	4-HOC_6_H_4_CHO	2i	50	90	186–188	188–190 (ref. 38)
10	4-Me_2_NC_6_H_4_CHO	2j	28	98	181–183	180–182 (ref. 39)

a Reaction conditions: aldehyde (1 mmol), malononitrile (1.05 mmol, 69 mg), 10 mg of NA-NHSO_3_H in 1 mL of water at room temperature.

Green chemistry emphasizes the development of new synthesis methods and biocompatible catalysts to prevent negative effects on human health and the environment. High atom economy, energy efficiency of processes, low amount of waste products, avoiding the formation of byproducts, renewable materials and replacing hazardous reagents or catalysts with less harmful materials are important and significant criteria in green chemistry. After completing the reaction, separating out the catalyst emphasizes ease of recovery, which is key in reducing waste and enhancing sustainability. Using crystallization with ethanol for product purification is a good choice, as ethanol is often regarded as a greener solvent compared to others, especially if it's derived from renewable sources. Knoevenagel and Claisen–Schmidt condensation reactions performed at room temperature using water align well with green chemistry principles, as they minimize energy consumption and use non-toxic solvents. The adoption of green chemistry principles during catalyst synthesis and product preparation indicates a commitment to reducing environmental impact. This includes minimizing hazardous substances, using renewable resources, and maximizing atom economy.

#### 3.2.2. Claisen–Schmidt condensation

A series of parameters were investigated to optimize the condensation reaction conditions of acetophenone (and ketones) with 4-chlorobenzaldehyde. These parameters included catalyst concentration, solvent nature, and reaction temperature. The best results for products 4a, 6a and 6c were achieved using 10 mg of NA-NHSO_3_H as the catalyst. These conditions were documented in entry 4 of Tables 4 and 5. The established conditions resulted in the highest yield and the shortest reaction time, outperforming other tested parameters. The results indicate that using 10 mg of NA-NHSO_3_H as a catalyst under the specified conditions significantly enhances the efficiency of the condensation reaction, making it a promising approach for synthesizing the desired products.

**Table 4:** Optimization of the Claisen–Schmidt condensation for the reaction of 4-chlorobenzaldehyde with acetophenone^a^

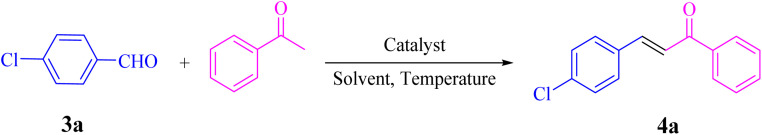					
Entry	NA-NHSO_3_H (mg)	Solvent	Temperature (°C)	Time (min)	Yield^b^ (%)
1	—	Water	r.t.	110	Trace
2	20	Water	r.t.	40	92
3	15	Water	r.t.	20	95
**4**	**10**	**Water**	**r.t.**	**20**	**97**
5	5	Water	r.t.	30	90
6	10	—	r.t.	65	81
7	10	Ethanol	r.t.	55	88
8	10	Water : ethanol (2 : 1)	r.t.	50	89
9	10	Tetrahydrofuran	r.t.	80	20
10	10	Water	45	35	87
11	10	Water	70	40	85
12	10	Water	r.t.	170	Trace^c^

a Reaction conditions: 4-chlorobenzaldehyde (1 mmol, 140 mg), acetophenone (1 mmol, 120 mg), NA-NHSO_3_H (mg) and solvent (1 mL).

b Isolated yield.

c The reaction catalyzed by NA-NH_2_.

**Table 5:** Optimization of the aldol condensation for the reaction of 4-chlorobenzaldehyde with ketones^a^

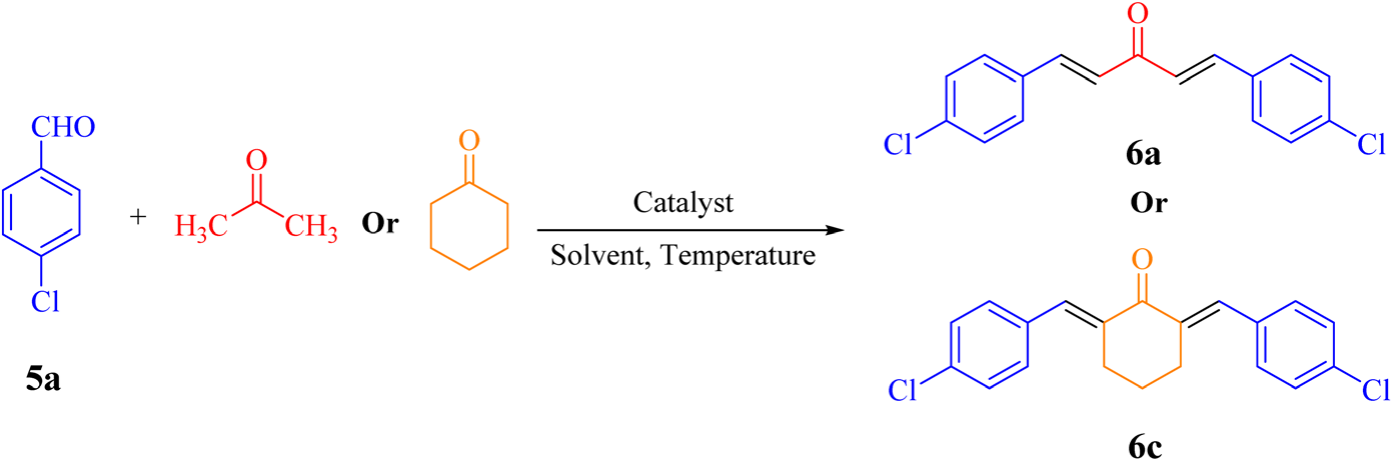							
Entry	NA-NHSO_3_H (mg)	Solvent	Temperature (°C)	Time (min)		Yield^b^ (%)	
				6a	6c	6a	6c
1	—	Water	r.t.	90	150	21	11
2	20	Water	r.t.	42	90	93	86
3	15	Water	r.t.	26	70	91	90
**4**	**10**	**Water**	**r.t.**	**28**	**50**	**96**	**96**
5	5	Water	r.t.	36	60	91	91
6	10	—	r.t.	68	72	80	84
7	10	Ethanol	r.t.	58	65	87	92
8	10	Water : ethanol (2 : 1)	r.t.	54	63	86	90
9	10	Toluene	r.t.	116	110	18	15
10	10	Water	45	44	58	83	85
11	10	Water	70	50	64	81	80
12	10	Water	r.t.	188	225	Trace^c^	Trace^c^

a Reaction conditions: 4-chlorobenzaldehyde (2 mmol, 240 mg), ketone (1 mmol, 58 mg), NA-NHSO_3_H (mg) and solvent (1 mL).

b Isolated yield.

c The reaction catalyzed by NA-NH_2_.

Solvents can significantly influence reaction kinetics, yield, and selectivity. Protic solvents, like water, often provide better results due to their ability to stabilize charged intermediates and facilitate proton transfer, which can enhance reaction rates and yields. In contrast, aprotic solvents (like tetrahydrofuran) and nonpolar solvents (like toluene) may not stabilize charged species as effectively, leading to longer reaction times and lower yields. This can be particularly important in reactions involving ionic intermediates or mechanisms that rely on hydrogen bonding (Tables 4 and 5).

According to the results, it was found that the use of protic solvents has a positive effect on the reaction and the selectivity towards the Knoevenagel and Claisen–Schmidt products is significantly affected. Because with decreasing temperature, the pH decreases, which increases the acidic strength and the reaction rate.

**Scheme 3:**
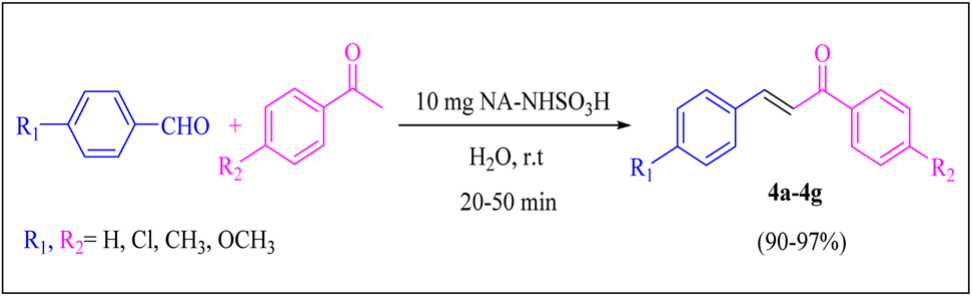
Claisen–Schmidt condensation of catalyzed by NA-NHSO_3_H.

In order to assess the generality of the catalytic protocol, the reaction was expanded to a wide range of aromatic aldehydes (Scheme 3). The outcomes are detailed in Table 6. The results indicate that the reactions proceeded cleanly, yielding the corresponding products with exceptional yields ranging from 90% to 97%. This high degree of yield across various substrates highlights the robustness and versatility of the NA-NHSO_3_H catalyst in facilitating the cyclocondensation process under the optimized conditions.

**Table 6:** NA-NHSO_3_H catalyzed the Claisen–Schmidt condensation in water at room temperature^a^

Entry	Aldehyde	Ketone	Product	Time (min)	Yield (%)	Mp (°C) Measured	Mp (°C) Literature
1	4-ClC_6_H_4_CHO	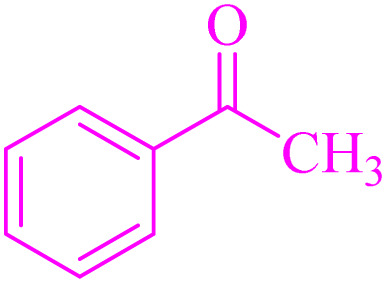	4a	20	97	110–112	110–112 (ref. 37)
2	C_6_H_5_CHO	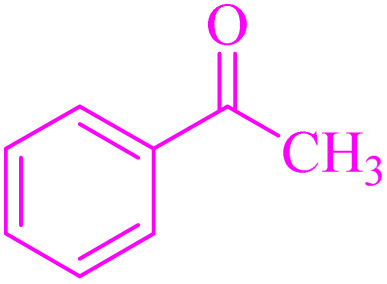	4b	28	96	50–52	54–56 (ref. 37)
3	C_6_H_5_CHO	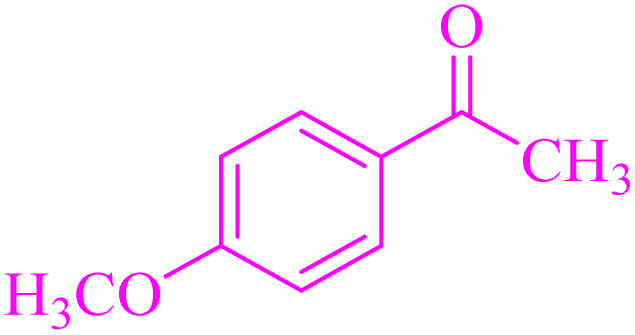	4c	34	93	104–106	102–105 (ref. 37)
4	4-ClC_6_H_4_CHO	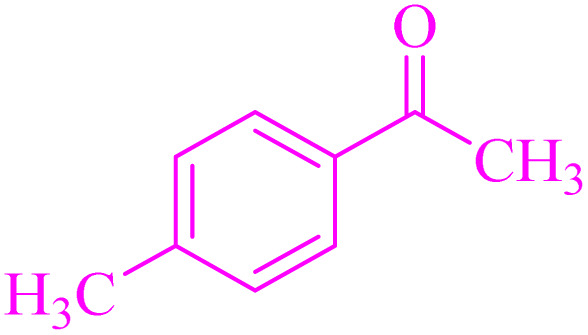	4d	42	91	144–146	143–144 (ref. 37)
5	4-ClC_6_H_4_CHO	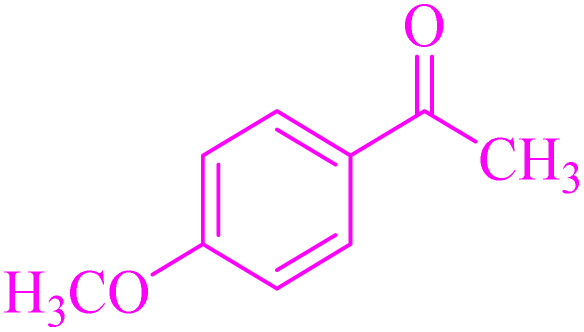	4e	36	93	120–124	125–126 (ref. 39)
6	4-ClC_6_H_4_CHO	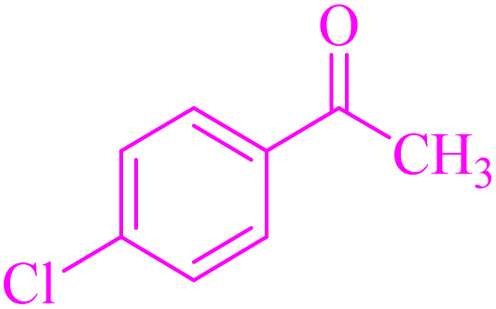	4f	34	94	150–152	148–150 (ref. 39)
7	4-MeOC_6_H_4_CHO	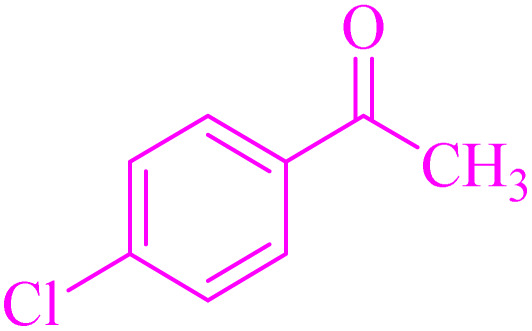	4g	50	90	118–121	119–120 (ref. 37)

a Reaction conditions: aldehyde (1 mmol), acetophenone (1 mmol, 120 mg), 10 mg of NA-NHSO_3_H in 1 mL of water at room temperature.

**Scheme 4:**
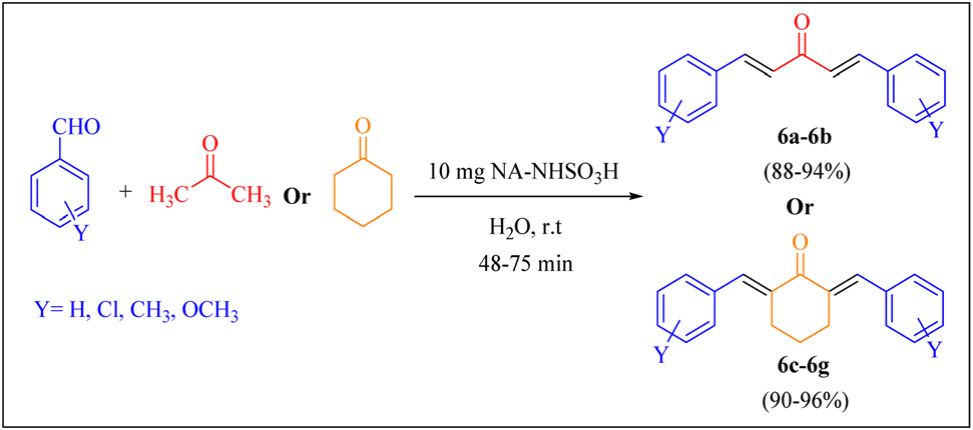
Synthesis of dibenzalacetone derivates and α,α′-bis(substituted-benzylidene) cycloalkanones from the reaction between various aldehydes with ketones catalyzed by NA-NHSO_3_H.

Also, the synthesis of electron-donating and electron-withdrawing aromatic aldehydes was studied using model reactions 6a and 6c (Scheme 4). The results showed that the desired products were synthesized with good to excellent yields regardless of the electronic nature of the substituent in the aldehydes. This suggests that the 6a and 6c model reactions accommodated a range of electron-donating and electron-withdrawing groups on the aromatic aldehydes, producing the desired products in satisfactory to high yields (Table 7).

**Table 7:** NA-NHSO_3_H catalyzed the Synthesis of α,β-unsaturated derivatives from the condensation between aldehydes with ketones^a^

Entry	Aldehyde	Ketone	Product	Time (min)	Yield (%)	Mp (°C) measured	Mp (°C) literature
1	C_6_H_5_CHO	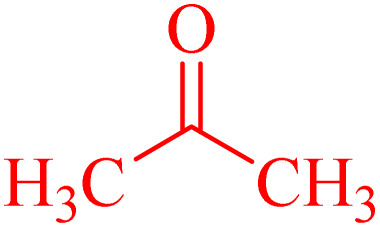	6a	48	94	40–42	41–43 (ref. 37)
2	4-MeC_6_H_4_CHO	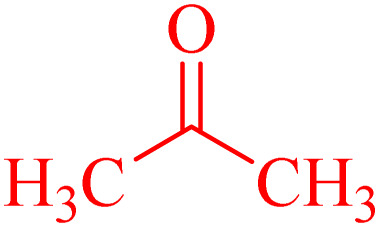	6b	55	88	122–124	126–129 (ref. 39)
3	4-ClC_6_H_4_CHO	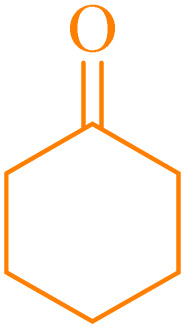	6c	50	96	144–146	145–147 (ref. 34)
4	4-MeC_6_H_4_CHO	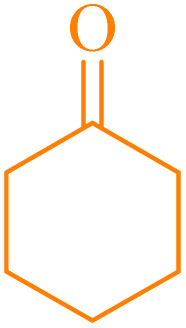	6d	70	93	199–201	199–200 (ref. 34)
5	4-MeOC_6_H_4_CHO	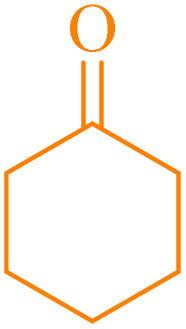	6e	75	90	168–170	169–171 (ref. 34)
6	C_6_H_5_CHO	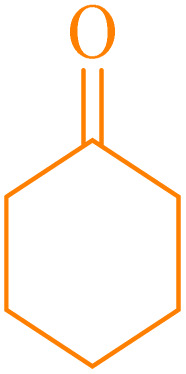	6f	60	95	116–118	115–117 (ref. 34)
7	3-O_2_NC_6_H_4_CHO	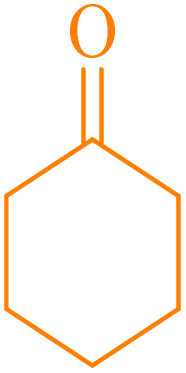	6g	80	91	198–200	200–202 (ref. 34)

a Reaction conditions: aldehyde (2 mmol), ketone (1 mmol, 58 mg), 10 mg of NA-NHSO_3_H in 1 mL of water at room temperature.

The reaction yields were calculated using eqn (3):3
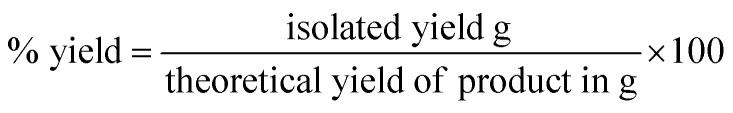


For further confirmation, ^13^CNMR, ^1^HNMR and FT-IR analysis were applied for some products and the results were displayed in Fig. S1–S21† which confirmed the formation of products.

### 3.3. Reusability of the catalyst leaching test

**Fig. 7:**
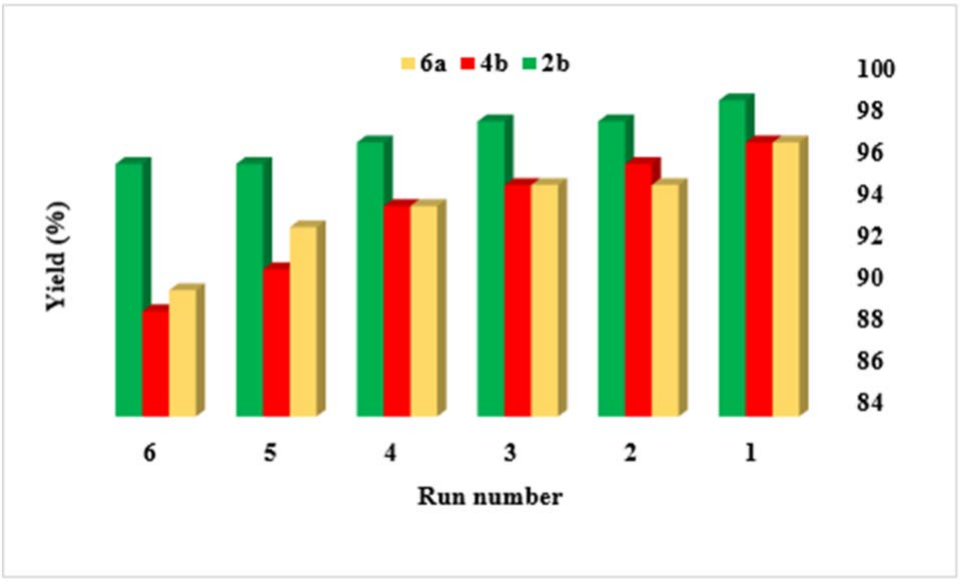
Reusability of NA-NHSO_3_H in the synthesis of products 2b, 4b and 6a.

**Fig. 8:**
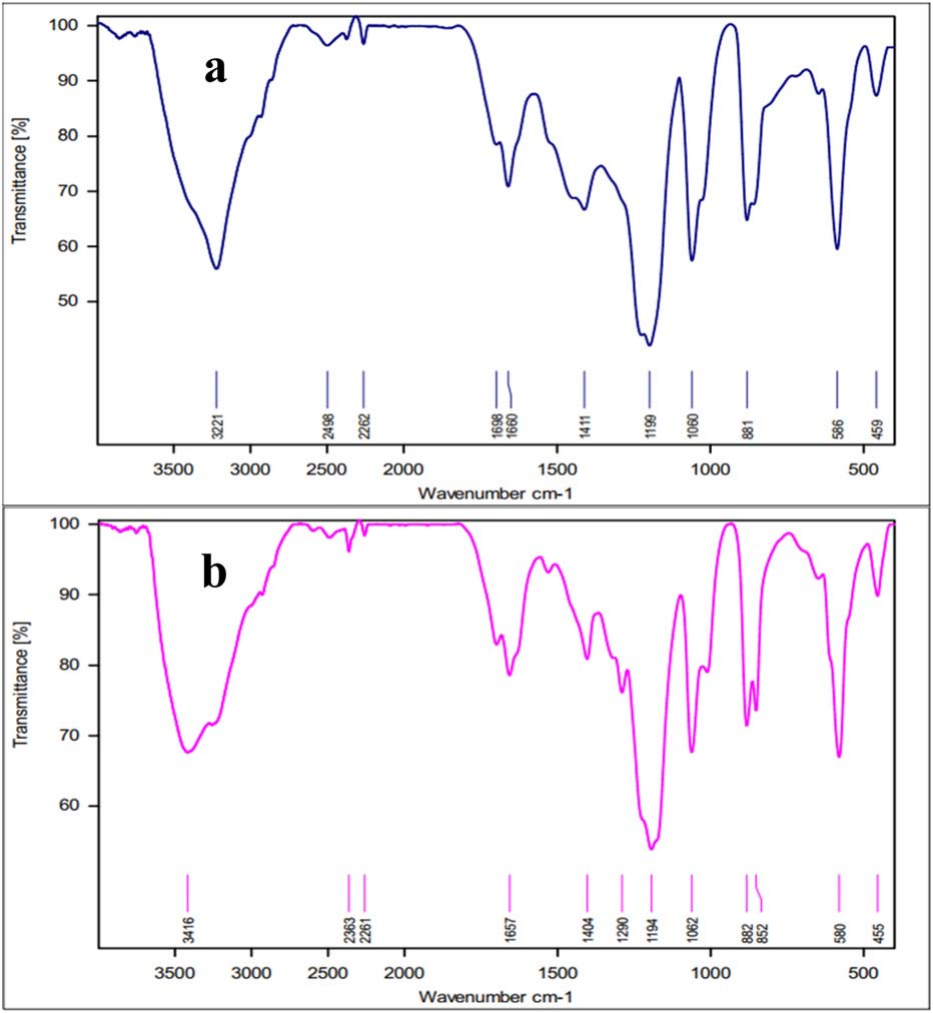
The FT-IR spectra of (a) fresh NA-NHSO_3_H and (b) NA-NHSO_3_H after recovery.

**Fig. 9:**
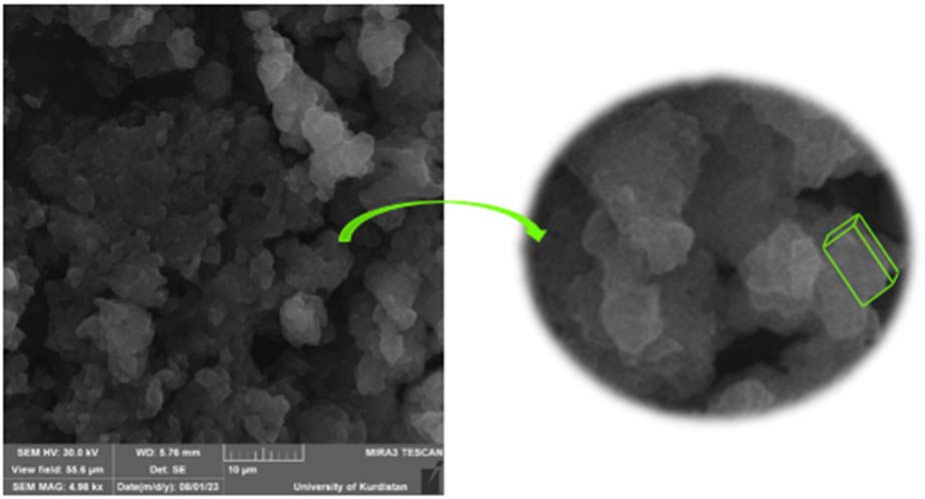
SEM images of NA-NHSO_3_H after recovery.

**Fig. 10:**
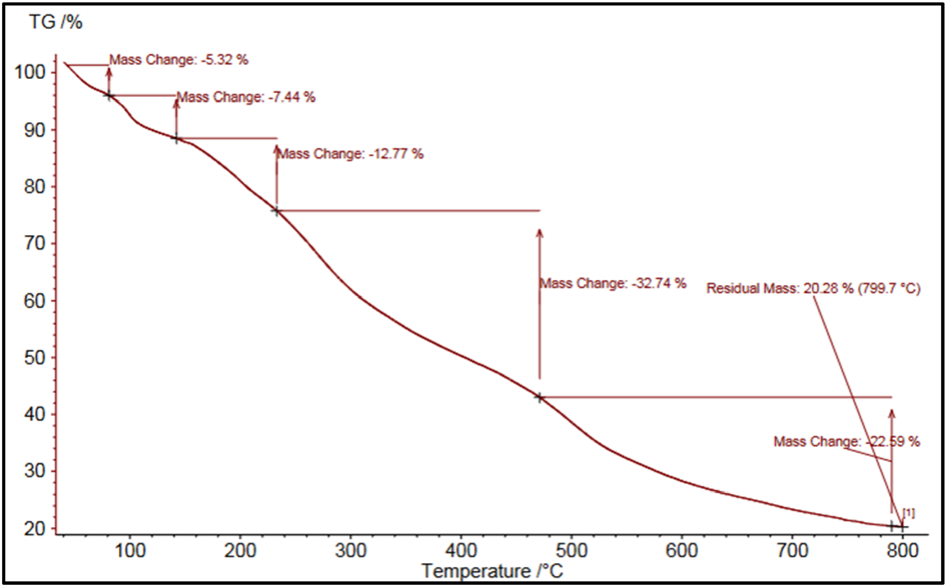
TG curve of NA-NHSO_3_H after recovery.

The reusability of catalysts is crucial for economic and environmental sustainability in catalytic processes. To investigate the reusability of this catalyst, reactions that produce products 2b, 4b, and 6a were evaluated (Fig. 7). After each cycle of the reaction, the catalyst can be easily recovered using hot ethanol, which minimizes loss and facilitates the reusability assessment. The recovered catalyst can then be washed and reused in subsequent reaction cycles. In summary, the catalytic activity was assessed over six cycles, demonstrating consistent performance with no significant loss in the original catalytic activity. Furthermore, The FT-IR spectra, SEM images and TG curves taken before and after recovery (Fig. 8–10) showed strong similarities, indicating that the catalyst maintained its stability throughout the testing. In addition, the solubility of the NA-NHSO_3_H catalyst before and after recovery in organic solvents including toluene, xylene and carbon disulfide was tested. The results confirmed that, unlike natural asphalt, NA-NHSO_3_H is not soluble in the mentioned solvents. Catalyst recovery studies shows a decline in product yield across successive cycles. It can be attributed to several factors. In the initial cycle, the catalyst typically displays its highest activity and selectivity, promoting efficient conversion to the desired product. Over subsequent cycles, catalyst deactivation, potentially due to structural changes or active site poisoning, leads to reduced selectivity and activity. Additionally, the accumulation of reactants, by-products, and residual solvents on catalyst can physically block active sites, thereby limiting access for reactants and reducing the overall catalytic activity.

The evaluation of leaching for the supported catalyst using a glass electrode indicates that there is no significant difference in the loading amount of acid groups on the surface of the catalyst even after six cycles. This suggests that the catalyst demonstrates good stability and retention of its –SO_3_H groups, which is crucial for maintaining its catalytic activity over multiple uses. The consistent loading of acid groups implies that the catalyst can be effectively reused without substantial loss of its active sites.

Dispersing 3 mg of the NA-NHSO_3_H catalyst in 3 mL of deionized water at room temperature is a standard method for assessing the acidity of solid catalysts. This ensures that the catalyst is adequately suspended for accurate pH measurement. The initial pH value of 2.02 indicates a strongly acidic environment, which is consistent with the presence of –SO_3_H groups on the catalyst. This acidity is crucial for the catalyst's performance in acid-catalyzed reactions. Continuing pH measurements for six more steps likely involves observing changes in pH over time with adding catalyst. This could help in understanding how the acidity of the catalyst evolves during the evaluation period. Monitoring pH can give insights into the stability of the acidic properties of the catalyst before and after recycling. Since acidity directly influences the catalytic activity of acid catalysts, maintaining low pH levels after recycling is desirable for consistent performance in catalytic reactions (Table 8).

**Table 8:** Investigation of the acidic effect of NA-NHSO_3_H before and after recovery

Entry	NA-NHSO_3_H		Reusability	
	Amount (mg)	pH	Run	pH
1	3	2.02	—	2.02
2	6	2.04	1	2.03
3	9	2.07	2	2.06
4	12	2.10	3	2.08
5	15	2.12	4	2.11
6	18	2.14	5	2.12
7	21	2.15	6	2.13

Comparison of the catalyst before and after recovery with NA indicates that the recovering process effectively eliminates excess ash and minerals from the catalyst. This is significant because impurities can affect the catalyst's performance, possibly leading to decreased activity and selectivity. The TG curves showing around 70% decomposition for the catalyst before recovery and 34% for NA imply that the recovery process not only enhances the purity of the catalyst but also significantly impacts its thermal stability. The higher decomposition rate in the catalyst suggests that it contained more volatile components or impurities initially. The reduction of these impurities likely contributes to improved catalytic performance since a purer catalyst is often more efficient. Removing unwanted materials can lead to better access to active sites for the reactants and minimize side reactions. The successful removal of ash and minerals through recovery processes could improve the recyclability of the catalyst, making it more economical for industrial applications. Ensuring high purity can help maintain consistent catalytic activity over multiple reaction cycles.

### 3.4. Hot filtration

The hot filtration test aimed to demonstrate the heterogeneous nature of the catalyst and to assess the leaching of acidic sites during the reaction. For this purpose, reaction 2a was conducted at room temperature in 1 mL of water. The catalyst was removed after 9 minutes, at which point the yield was 57%. The reaction mixture was stirred for an additional 9 minutes without the catalyst, resulting in a final yield of 60%. The catalyst removal showed that the reaction yielded 57% in the presence of the catalyst. The stability of the –SO_3_H groups on the NA support surface has been confirmed. This confirms that the –SO_3_H groups are covalently bonded to the catalytic support material. The results show that the acidic –SO_3_H groups are not leaching into the reaction solution. Additionally, the –SO_3_H groups are not degrading under the reaction conditions. The stability of the covalent attachment of the –SO_3_H groups and the lack of leaching are significant findings. This indicates that the catalyst design is effective at maintaining the integrity of the active sites and preventing contamination of the reaction mixture. These results suggest the catalyst has good long-term stability and recyclability, which are important practical considerations for industrial applications. The covalent bonding also likely improves the overall performance and efficiency of the catalytic system. The data supports the heterogeneous nature of the catalyst, as significant leaching was not observed. This allows for potential reusability without significant loss of active sites.

### 3.5. Comparison

The study compares NA-NHSO_3_H with various reported catalysts for specific reactions (2b and 4b) in the Knoevenagel and Claisen–Schmidt condensations. NA-NHSO_3_H can function effectively at lower temperatures and pressures, which is crucial for reactions involving sensitive substrates that might degrade or react undesirably under harsher conditions (milder reaction conditions). The catalyst enables faster reactions, which can increase efficiency in synthetic processes. The catalyst demonstrated superior performance in terms of yield compared to other catalysts, indicating its effectiveness (Table 9).

**Table 9:** Comparison of activity of NA-NHSO_3_H with previous reports

Entry	Catalyst	Conditions	Yield (%)	Product	[Ref.]
1	Imine-linked COFs (TaDA)	12 h/100 °C, toluene	97	2b	40
2	Zn/Cd-metal–organic frameworks	6 h/60 °C	99	2b	41
3	Calix[4]arene-based polyoxometalate	1 h/60 °C	99	2b	42
4	Li-zagronas	90 min/r.t., ethanol	90	2b	39
5	Chitosan	6 h/40 °C, ethanol	99	2b	43
6	BCN	30 min/80 °C, acetonitrile	92	2b	44
7	NA-NHSO_3_H	24 min/r.t., water	96	2b	This work
8	Cs-zagronas	30 min/r.t., ethanol	95	4b	39
9	GO	16 h/100 °C, TBAB, solvent-free	84	4b	45
10	K-NAS	210 min/r.t., ethanol	95	4b	37
12	NA-NHSO_3_H	28 min/r.t., water	96	4b	This work

The characteristics of NA-NHSO_3_H position it as a user-friendly and environmentally benign catalyst, aligning with the principles of green chemistry. This is particularly important in modern chemistry, where sustainability and reduced environmental impact are priorities. The superior performance of NA-NHSO_3_H suggests it may be a more viable option for industrial applications or in laboratories seeking efficient and eco-friendly methodologies.

## 4. Conclusion

In summary, a metal-free solid acid catalyst, NA-NHSO_3_H, has been introduced for the synthesis of α,β-unsaturated compounds, dibenzalacetone derivatives and α,α′-bis(substituted-benzylidene) cycloalkanones. Notable advantages of this protocol are good to excellent yields, rapid reaction times, simplified one-pot synthesis, use of environmentally-friendly “green” reaction conditions, reduced number of synthetic steps, minimal waste generation, easy product separation, good reaction conditions and low operational costs. Additionally, the catalyst demonstrates excellent reusability without significant loss of activity. These are all highly desirable features for an efficient and sustainable chemical process or synthesis. The combination of high productivity, resource efficiency and cost-effectiveness could make this a very attractive approach. The recovered catalyst was characterized using FT-IR, SEM and TGA techniques, confirming its structural integrity and performance.

## Data availability

The data that support the findings of this study are available in ESI.†

## Author contributions

Sahar Abdolahi: validation, methodology, investigation, writing – original draft, conceptualization. Mohammad Soleiman-Beigi: funding acquisition, supervision, project administration, conceptualization, editing, resources.

## Conflicts of interest

All authors declare that there are no competing interests.

## Supplementary Material

RA-015-D5RA00870K-s001
