# Supplementary material for: Exploring the catalytic activity of –NHSO3H functionalized natural asphalt: a sustainable and efficient catalyst for condensation reactions in water
Source: RSC Adv. 2025 Mar 26;15(12):9280–94. doi: 10.1039/d5ra00870k (PMC11939071; doi:10.1039/d5ra00870k)
Supplement: RA-015-D5RA00870K-s001 [file RA-015-D5RA00870K-s001.pdf]

Supplementary Information (SI) for RSC Advances.
This journal is © The Royal Society of Chemistry 2025

Supporting information

Exploring the Catalytic Activity of –$NHSO_3H$ Functionalized Natural Asphalt: A Sustainable and Efficient Catalyst for Condensation Reactions in Water

Sahar Abdolahi [a], Mohammad Soleiman-Beigi [a,*]

[a] *Department of Chemistry, Faculty of Basic Sciences, Ilam University, P.O. Box 69315516, Ilam, Iran.*

[*] Corresponding author.
E-mail addresses: SoleimanBeigi@yahoo.com; m.soleimanbeigi@ilam.ac.ir.

Spectral data of some of the products:

2-(4-chlorobenzylidene)malononitrile (Figs S1-S3, Table 3, 2a): Melting point: 160-162 °C. FT-IR (KBr) (v_{max}/cm^{-1}): 1406-1643, 2358 cm^{-1}. ^{1}H NMR (400 MHz, $CDCl_3$): δ (ppm)= 7.69 (d, *J* = 8.2 Hz, 4H), 7.93 (d, *J* = 8.2 Hz, 4H), 8.51 (s, 2H). ^{13}C NMR (100 MHz, $CDCl_3$): δ (ppm)= 82.7, 113.4, 114.4, 130.1, 132.5, 139.4, 160.5.

2-benzylidenemalononitrile (Figs S4-S6, Table 4, 2b): Melting point: 80 °C. FT-IR (KBr) (v_{max}/cm^{-1}): 1449-1640, 2225 cm^{-1}. ^{1}H NMR (400 MHz, $CDCl_3$): δ (ppm)= 7.57 (m, 3H), 7.67 (d, *J* = 8 Hz, 2H), 7. 82 (s, 1H). ^{13}C NMR (100 MHz, $CDCl_3$): δ (ppm)= 82.8, 112.6, 128.5, 130.2, 130.8, 134.7, 160.1.

2-(4-(dimethylamino) benzylidene) malononitrile (Figs S7-S9, Table 3, 2j): Melting point: 181-183 °C. FT-IR (KBr) (v_{max}/cm^{-1}): 1441-1614, 2208 cm^{-1}. ^{1}H NMR (400 MHz, $CDCl_3$): δ (ppm)= 6.84 (d, *J* = 8 Hz, 2H), 7.82 (d, *J* = 8 Hz, 2H), 8. 03 (s, 1H). ^{13}C NMR (100 MHz, $CDCl_3$): δ (ppm)= 39.6, 68.6, 111.9, 115.5, 116.2, 118.7, 133.5, 154.3,158.8.

(E)-chalcone (Figs S10-S12, Table 7, 4b): Melting point: 50-52 °C. FT-IR (KBr) (v_{max}/cm^{-1}): 1445–1603, 1662, 2855-2924 cm^{-1}. ^{1}H NMR (400 MHz, $CDCl_3$): δ (ppm)= 7.43 (d, *J* = 8 Hz, 2H), 7. 53 (d, *J* = 8 Hz, 2H), 7.56 (d, *J* = 16 Hz, 1H), 7.62 (d, *J* = 4 Hz, 2H), 7.86 (d, *J* = 16 Hz, 1H), 8.05 (d, *J* = 8 Hz, 2H). ^{13}C NMR (100 MHz, $CDCl_3$): δ (ppm)= 122.08, 128.5, 128.7, 129.02, 130.6, 132.8, 134.9, 138.2, 144.8, 190.5.

(E)-1-(4-methoxyphenyl)-3-phenylprop-2-en-1-one (Figs S13-S15, Table 6, 4c): Melting point: 104-106 °C. FT-IR (KBr) (v_{max}/cm^{-1}): 1439- 1603, 1653, 2850-2926 cm^{-1}. ^{1}H NMR (400 MHz, $CDCl_3$): δ (ppm)= 2.92(s, 3H), 7.45 (d, *J* = 8 Hz, 2H), 7.58 (d, *J* = 16 Hz, 1H), 7. 67 (m, 2H), 7.84 (d, *J* = 16 Hz, 1H) 8.07 (d, *J* = 8 Hz, 2H). ^{13}C NMR (100 MHz, $CDCl_3$): δ (ppm)= 55.5, 113.8, 121.8, 128.4, 128.9, 130.3, 130.8, 131.1, 135.1, 144.0, 163.4, 188.7.

(E)-3-(4-chlorophenyl)-1-(p-tolyl)prop-2-en-1-one (Figs S16-S18, Table 6, 4d): Melting point: 144-146 °C. FT-IR (KBr) (v_{max}/cm^{-1}): 1406–1603, 1656, 2856-2921 cm^{-1}. ^{1}H NMR (400 MHz, $CDCl_3$): δ (ppm)= 2.47(s, 3H), 7.33 (d, *J* = 8 Hz, 2H), 7. 42 (d, *J* = 8 Hz, 2H), 7.56 (d, *J* = 16 Hz, 1H), 7.60 (d, *J* = 4 Hz, 2H), 7.80 (d, *J* = 16 Hz, 1H), 7.97 (d, *J* = 8 Hz, 2H). ^{13}C NMR (100 MHz, $CDCl_3$): δ (ppm)= 21.7, 122.4, 128.6, 129.2, 129.4, 129.5, 133.15, 135.4, 136.3, 142.9,143.8, 189.7.

(1E,4E)-1,5-diphenylpenta-1,4-dien-3-one (Figs S19-S21, Table 7, 6a): Melting point: 40-42 °C. FT-IR (KBr) (v_{max}/cm^{-1}): 1447–1622, 1651, 3026-3054 cm^{-1}. ^{1}H NMR (400 MHz, $CDCl_3$): δ (ppm)= 7.21 (dd, *J* = 8 Hz, 2H), 7.33 (d, *J* = 8 Hz, 2H), 7. 34 (dd, *J* = 8 Hz, 2H), 7.38 (*J* = 16 Hz), 7.45 (*J* = 4 Hz, 2H), 7.65 (*J* = 16 Hz, 1H), 7.80 (*J* = 8 Hz, 2H). ^{13}C NMR (100 MHz, $CDCl_3$): δ (ppm)= 125.4, 128.4, 128.8, 130.5, 134.8, 143.3, 188.9.

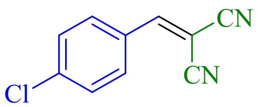

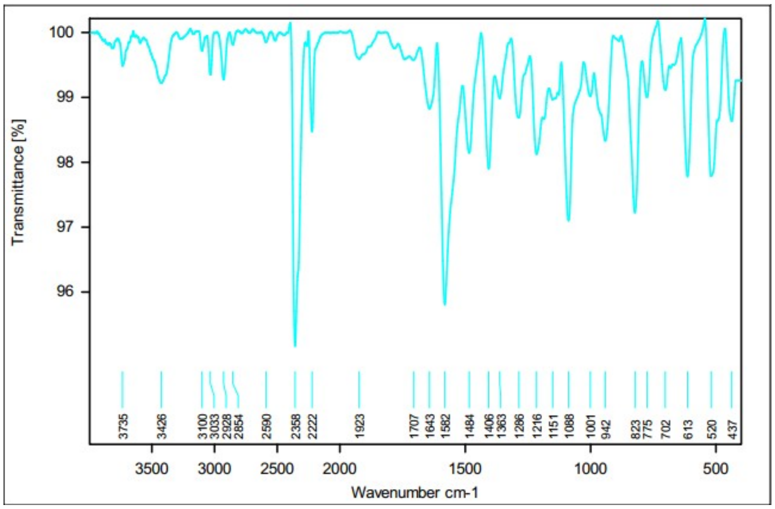

Fig. S1 IR spectrum of 2-(4-chlorobenzylidene)malononitrile

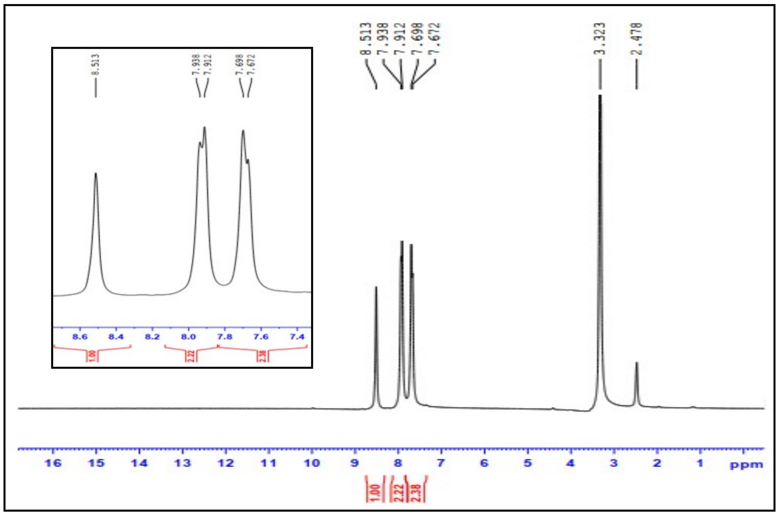

Fig. S2 ^{1}H NMR spectrum of 2-(4-chlorobenzylidene)malononitrile

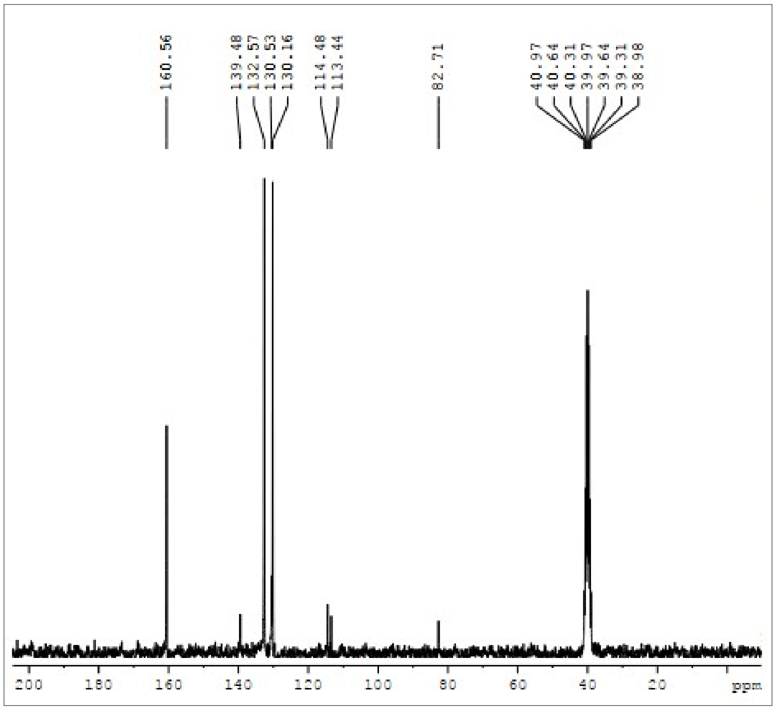

Fig. S3 ^{13}C NMR spectrum of 2-(4-chlorobenzylidene)malononitrile

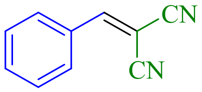

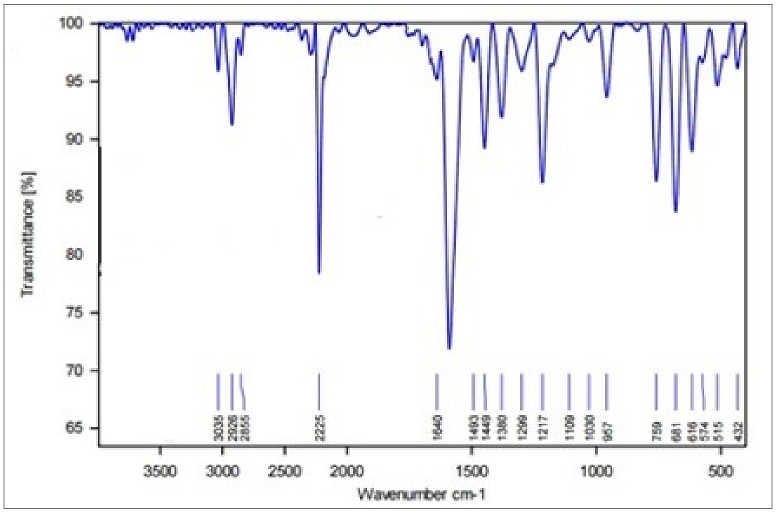

Fig. S4 IR spectrum of 2-benzylidenemalononitrile

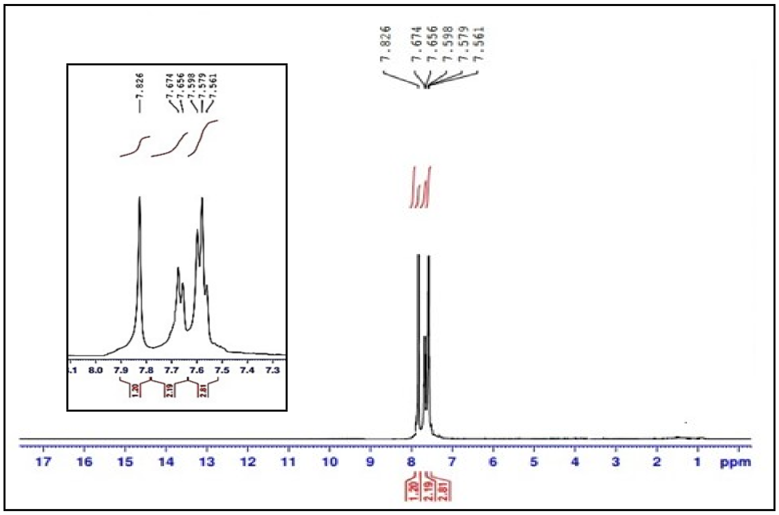

Fig. S5 ^{1}H NMR spectrum of 2-benzylidenemalononitrile

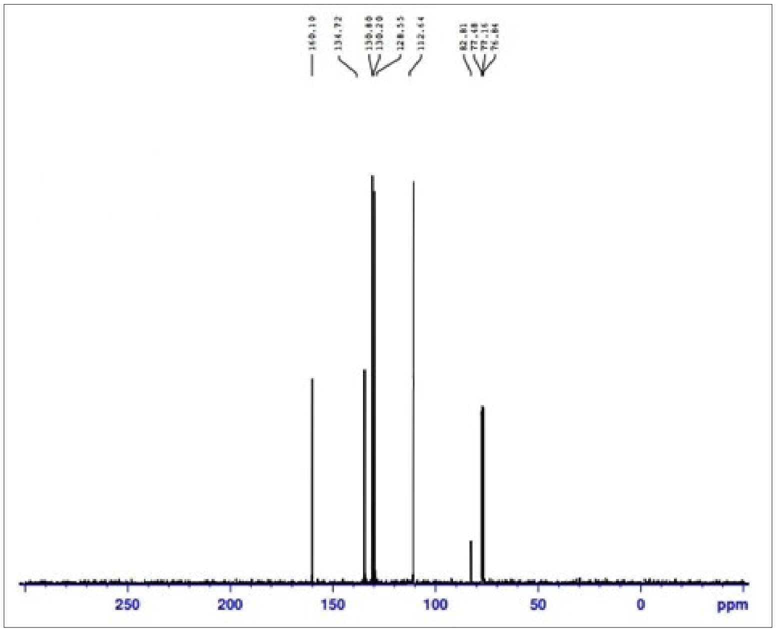

Fig. S6 ^{13}C NMR spectrum of 2-benzylidenemalononitrile

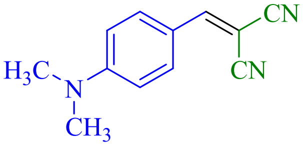

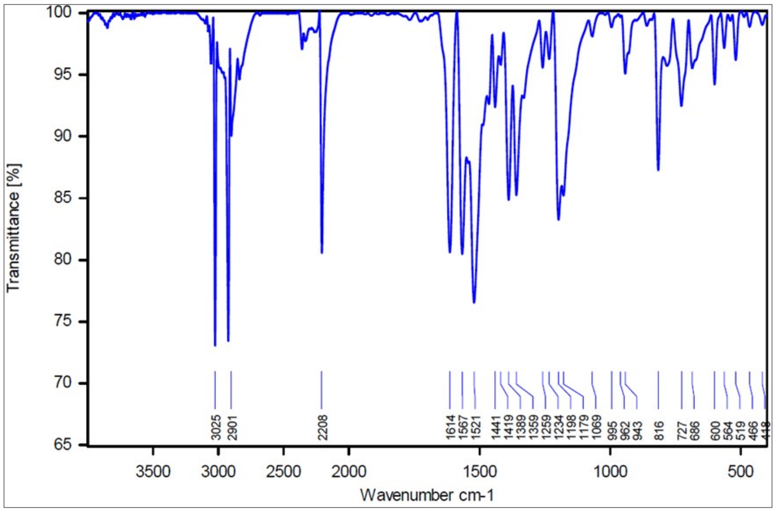

Fig. S7 IR spectrum of 2-(4-(dimethylamino)benzylidene)malononitrile

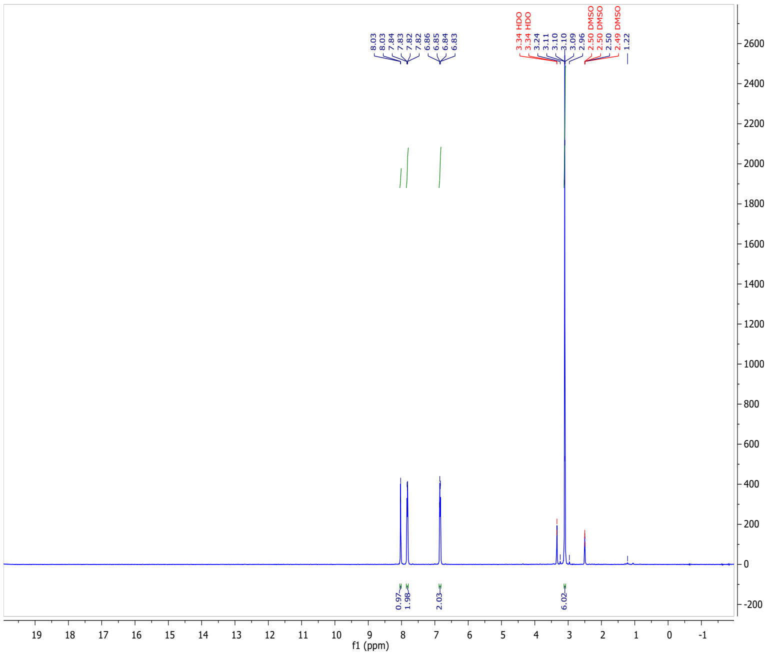

Fig. S8 ^{1}H NMR spectrum of 2-(4-(dimethylamino)benzylidene)malononitrile

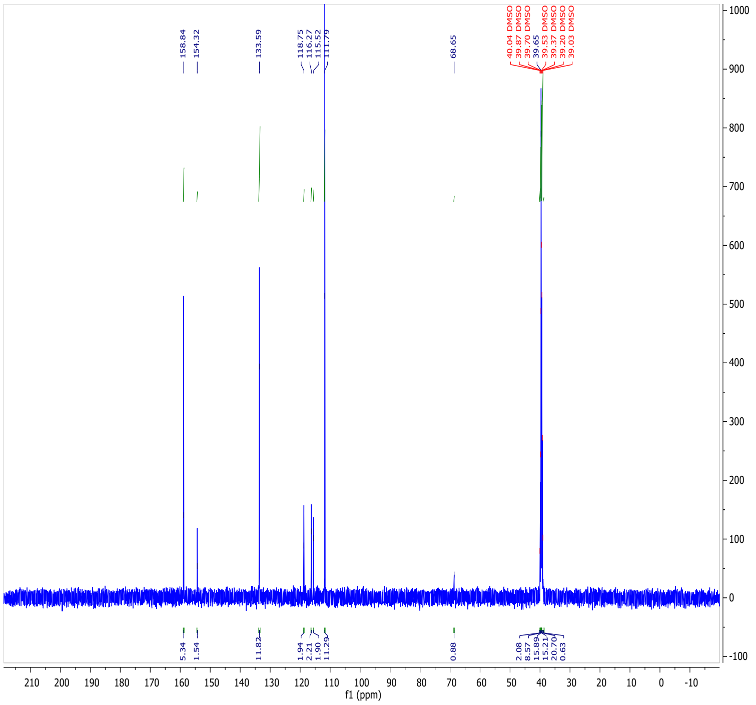

Fig. S9 ^{13}C NMR spectrum of 2-(4-(dimethylamino)benzylidene)malononitrile

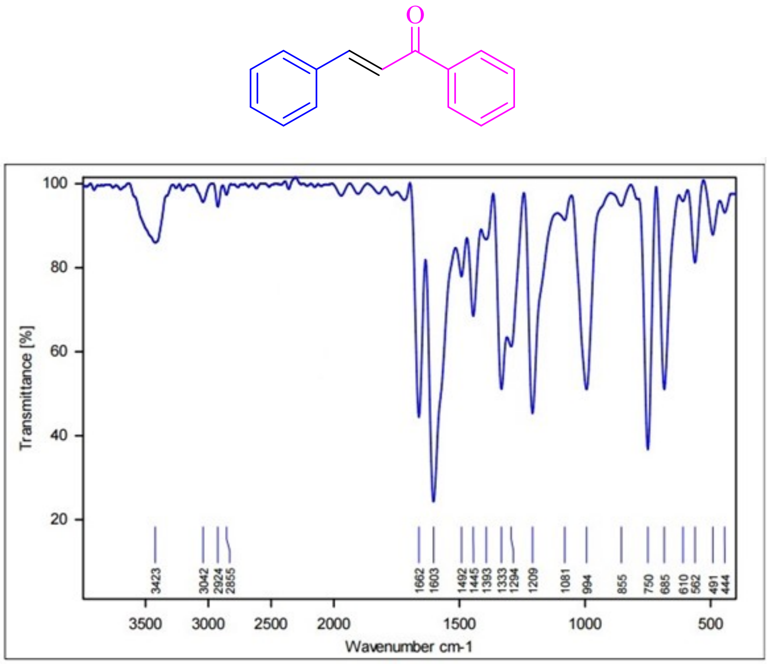

Fig. S10 IR spectrum of (E)-chalcone

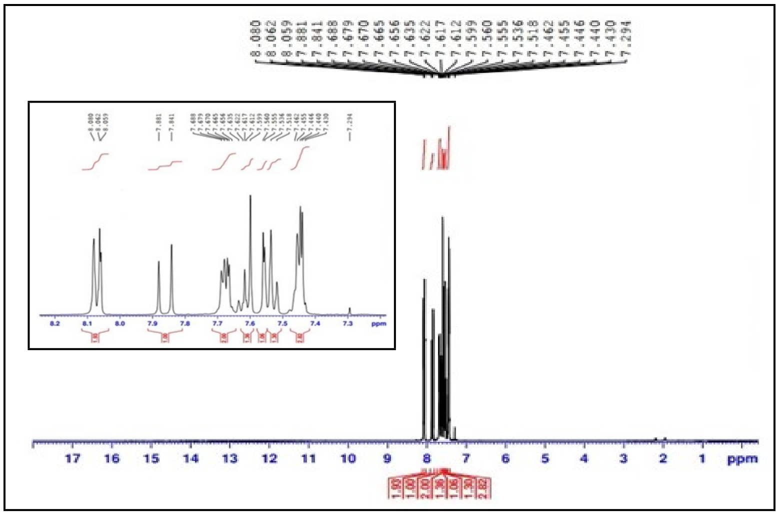

Fig. S11 ^{1}H NMR spectrum of (E)-chalcone

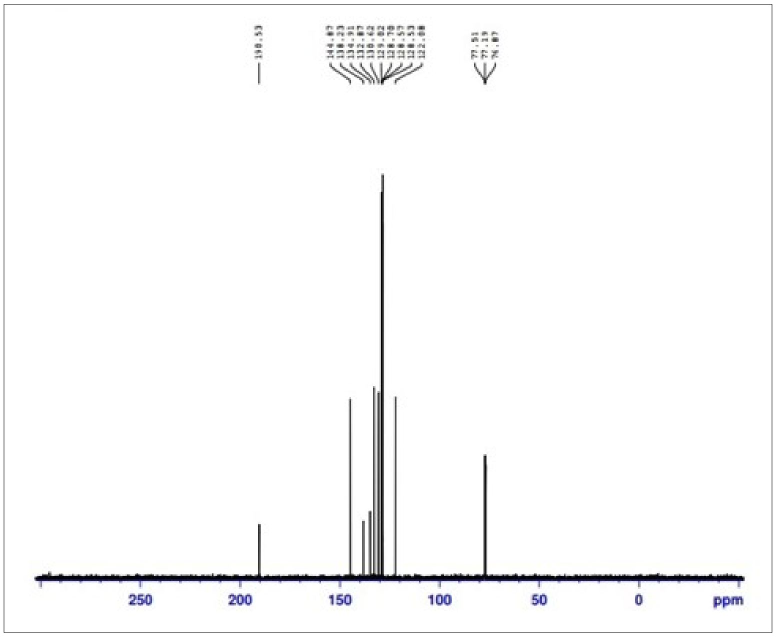

Fig. S12 ^{13}C NMR spectrum of (E)-chalcone

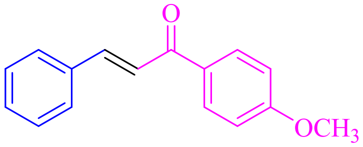

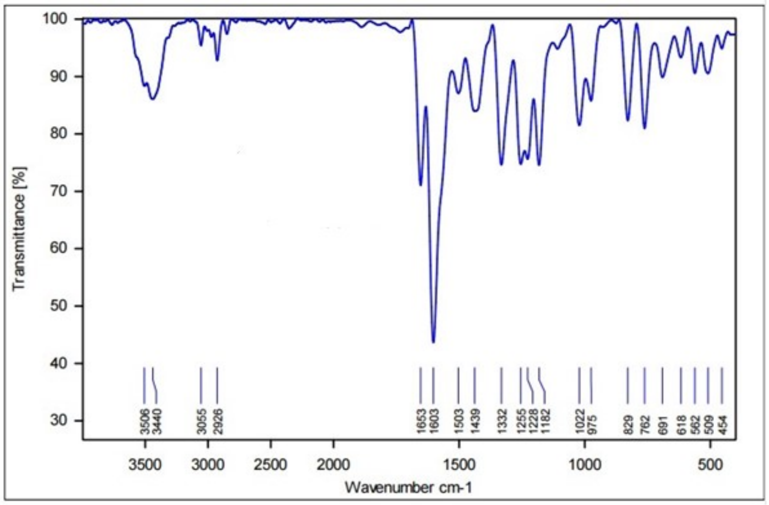

Fig. S13 IR spectrum of (E)-1-(4-methoxyphenyl)-3-phenylprop-2-en-1-one

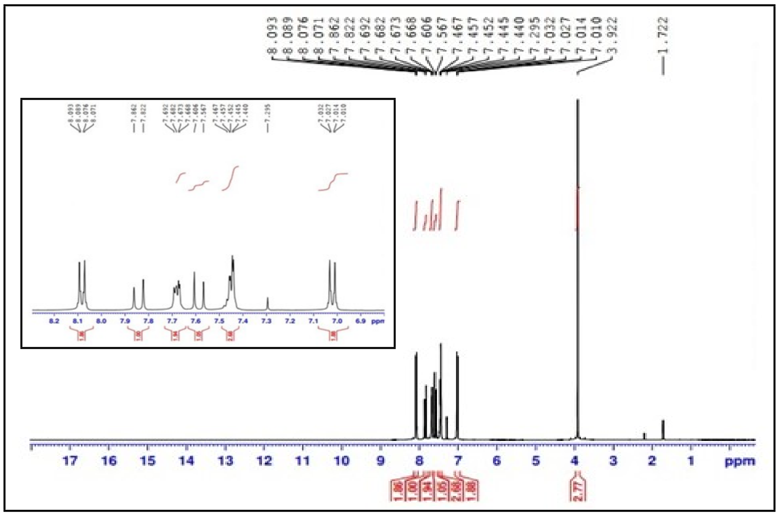

Fig. S14 ^{1}H NMR spectrum of (E)-1-(4-methoxyphenyl)-3-phenylprop-2-en-1-one

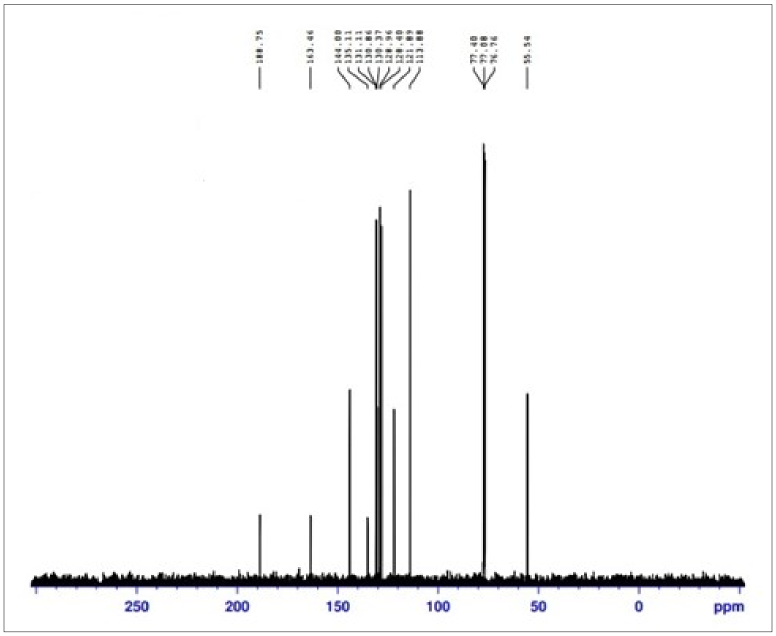

Fig. S15 ^{13}C NMR spectrum of (E)-1-(4-methoxyphenyl)-3-phenylprop-2-en-1-one

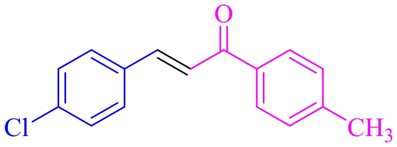

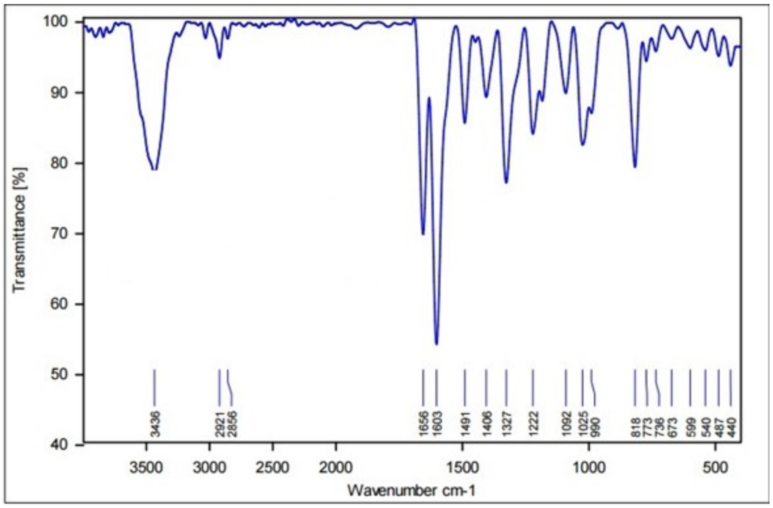

Fig. S16 IR spectrum of (E)-3-(4-chlorophenyl)-1-(p-tolyl)prop-2-en-1-one

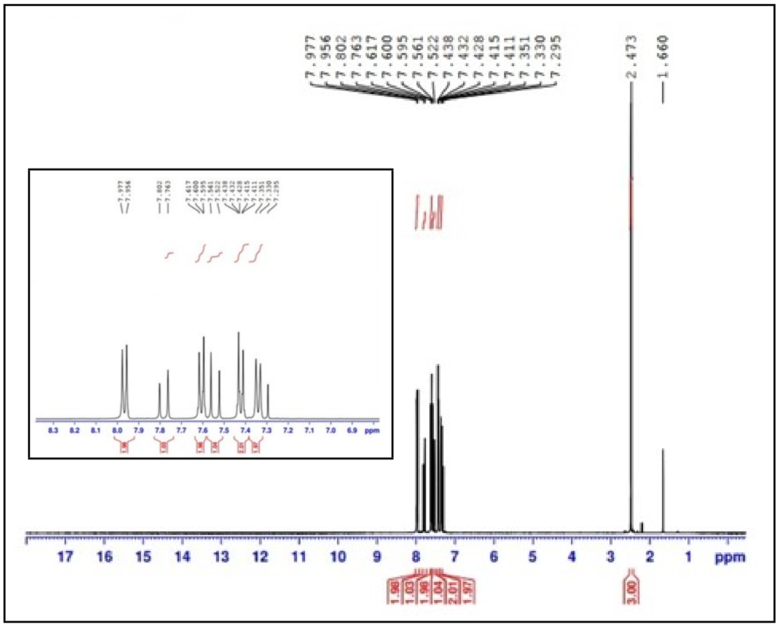

Fig. S17 ^{1}H NMR spectrum of (E)-3-(4-chlorophenyl)-1-(p-tolyl) prop-2-en-1-one

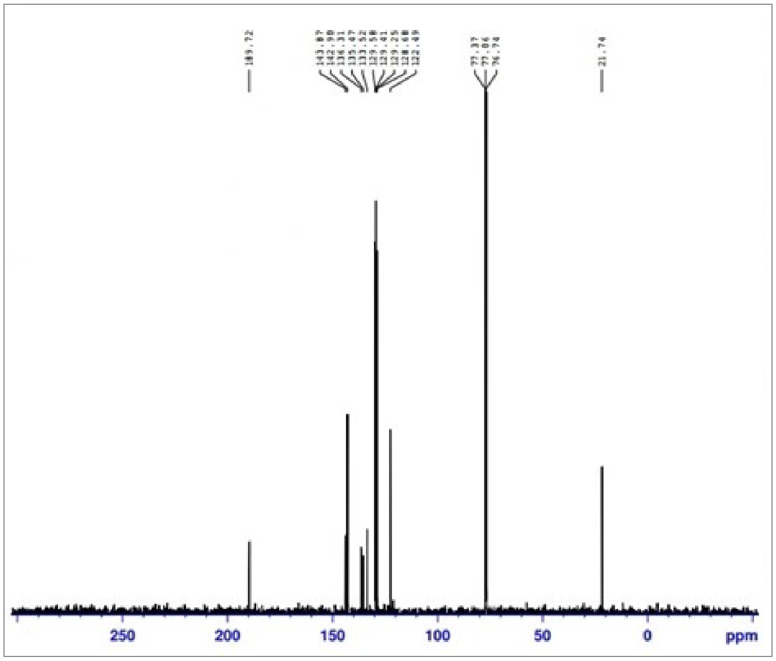

Fig. S18 ^{13}C NMR spectrum of (E)-3-(4-chlorophenyl)-1-(p-tolyl) prop-2-en-1-one

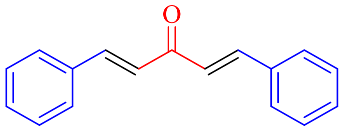

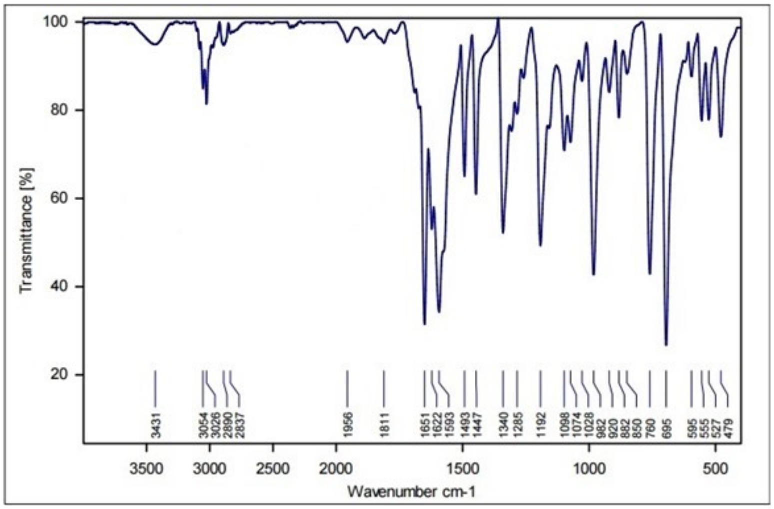

Fig. S19 IR spectrum of (1E,4E)-1,5-diphenylpenta-1,4-dien-3-one

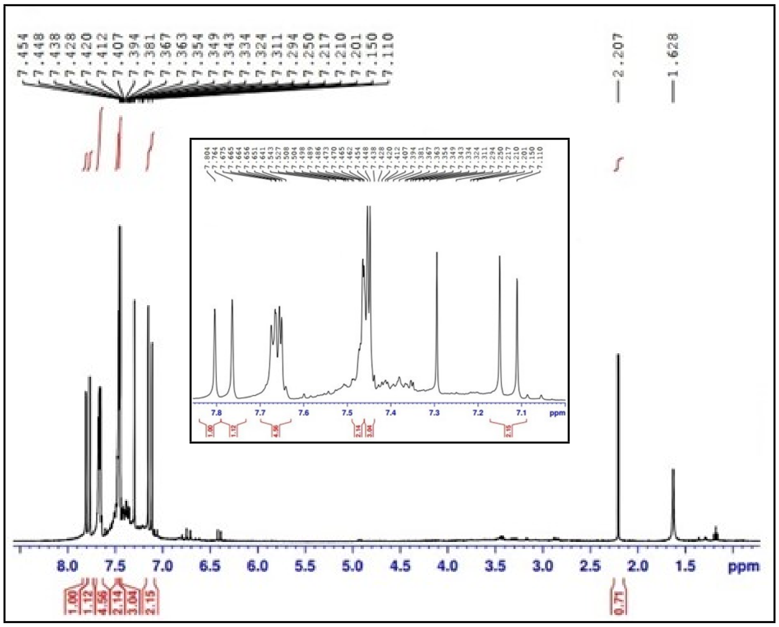

Fig. S20 ^{1}H NMR spectrum of (1E,4E)-1,5-diphenylpenta-1,4-dien-3-one

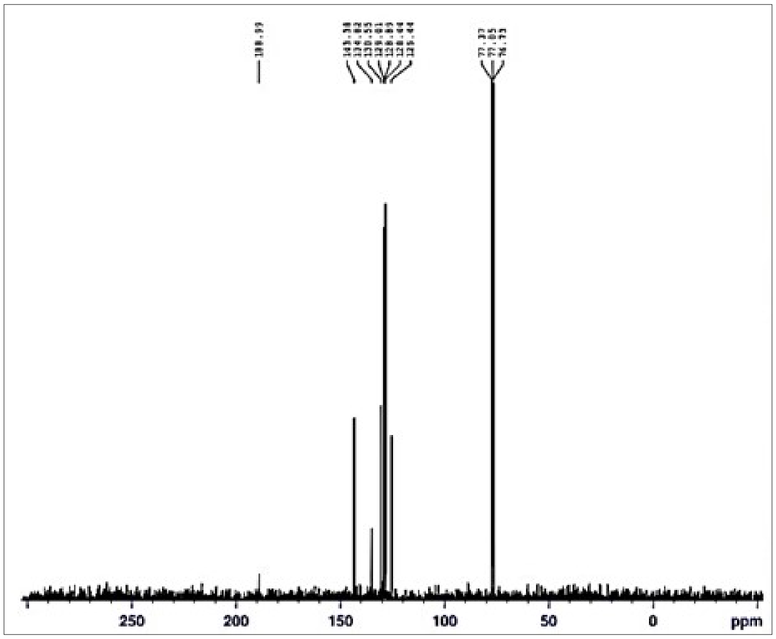

Fig. S21 ^{13}C NMR spectrum of (1E,4E)-1,5-diphenylpenta-1,4-dien-3-one
